# Hemichannel-Mediated and pH-Based Feedback from Horizontal Cells to Cones in the Vertebrate Retina

**DOI:** 10.1371/journal.pone.0006090

**Published:** 2009-06-30

**Authors:** Iris Fahrenfort, Marvin Steijaert, Trijntje Sjoerdsma, Evan Vickers, Harris Ripps, Jorrit van Asselt, Duco Endeman, Jan Klooster, Robert Numan, Huub ten Eikelder, Henrique von Gersdorff, Maarten Kamermans

**Affiliations:** 1 Research Unit Retinal Signal Processing, The Netherlands Institute for Neuroscience, Amsterdam, The Netherlands; 2 Department of Neurogenetics, Academic Medical Center, University of Amsterdam, Amsterdam, The Netherlands; 3 Department of Medical Physics, Academic Medical Center, University of Amsterdam, Amsterdam, The Netherlands; 4 Department of Biomedical Engineering, Biomodeling and Bioinformatics, Eindhoven University of Technology, Eindhoven, The Netherlands; 5 Vollum Institute, Oregon Health & Science University, Portland, Oregon, United States of America; 6 Department of Ophthalmology and Visual Sciences, University of Illinois at Chicago, Chicago, Illinois, United States of America; 7 Marine Biological Laboratory, Woods Hole, Massachusetts, United States of America; Mount Sinai School of Medicine, United States of America

## Abstract

**Background:**

Recent studies designed to identify the mechanism by which retinal horizontal cells communicate with cones have implicated two processes. According to one account, horizontal cell hyperpolarization induces an increase in pH within the synaptic cleft that activates the calcium current (Ca^2+^-current) in cones, enhancing transmitter release. An alternative account suggests that horizontal cell hyperpolarization increases the Ca^2+^-current to promote transmitter release through a hemichannel-mediated ephaptic mechanism.

**Methodology/Principal Findings:**

To distinguish between these mechanisms, we interfered with the pH regulating systems in the retina and studied the effects on the feedback responses of cones and horizontal cells. We found that the pH buffers HEPES and Tris partially inhibit feedback responses in cones and horizontal cells and lead to intracellular acidification of neurons. Application of 25 mM acetate, which does not change the extracellular pH buffer capacity, does lead to both intracellular acidification and inhibition of feedback. Because intracellular acidification is known to inhibit hemichannels, the key experiment used to test the pH hypothesis, i.e. increasing the extracellular pH buffer capacity, does not discriminate between a pH-based feedback system and a hemichannel-mediated feedback system. To test the pH hypothesis in a manner independent of artificial pH-buffer systems, we studied the effect of interfering with the endogenous pH buffer, the bicarbonate/carbonic anhydrase system. Inhibition of carbonic anhydrase allowed for large changes in pH in the synaptic cleft of bipolar cell terminals and cone terminals, but the predicted enhancement of the cone feedback responses, according to the pH-hypothesis, was not observed. These experiments thus failed to support a proton mediated feedback mechanism. The alternative hypothesis, the hemichannel-mediated ephaptic feedback mechanism, was therefore studied experimentally, and its feasibility was buttressed by means of a quantitative computer model of the cone/horizontal cell synapse.

**Conclusion:**

We conclude that the data presented in this paper offers further support for physiologically relevant ephaptic interactions in the retina.

## Introduction

Feedback mechanisms in neural systems provide pathways for reciprocal actions of pre- and post-synaptic cells. One of the best documented examples of feedback is that which governs the center-surround receptive field organization of retinal neurons. There is abundant evidence that the earliest stage at which feedback occurs is at the first synapse in the visual pathway, i.e., between the axon terminals of photoreceptors and the dendritic processes of horizontal cells both for cones [1]–[5] and for rods [6]. Although extensively investigated in many vertebrate species, there is still no general agreement as to the mechanism that mediates feedback. Nevertheless, two appealing, but very different, views on how feedback modulates the Ca^2+^-current in cones have emerged from recent studies on lower vertebrates.

**Figure 1:**
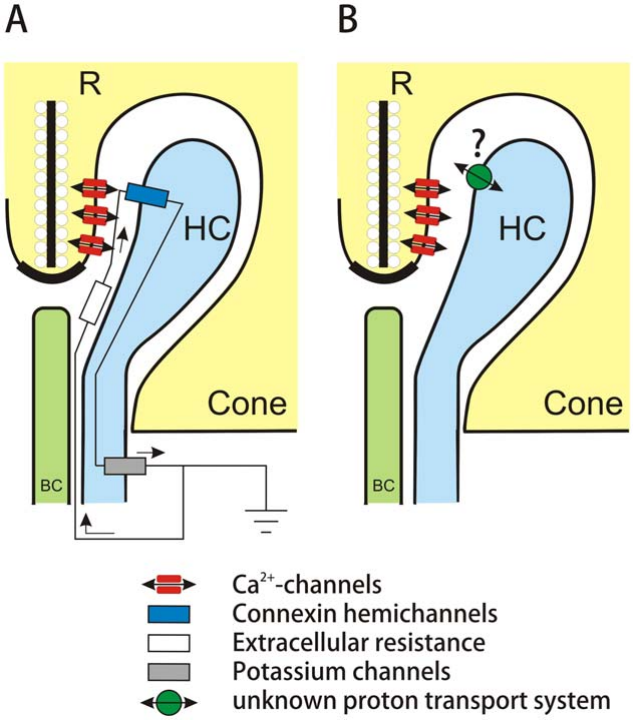
Schemeatic representation of the two feedback hypothesis. A) the ephaptic hypothesis and B) the pH hypothesis. The cone terminal is depicted with the invaginating HC and BC dendrites. The synaptic ribbon with the synaptic vesicles is indicated by “R”. The voltage gated Ca-channels in the cone synaptic terminal are depicted in red. Hemichannels in the HC dendrites are indicated by the blue symbol. The white symbol indicate the extracellular resistance and the gray symbol the potassium channels in the HCs. The green symbol indicates an unidentified proton transport system.

Kamermans et al. [7] have proposed that hemichannels (connexons) on horizontal cell dendrites that invade photoreceptor synaptic terminals, voltage-activated Ca^2+^-channels in cones, and glutamate-gated channels on horizontal cells act in concert through an ephaptic mechanism to modulate the Ca^2+^-current in goldfish cones and thereby regulate transmitter release (Fig. 1A). An ephaptic mechanism functioning in the cone synapse was first proposed by Byzov and Shura-Bura [8]. According to the hemichannel hypothesis, surround illumination causes the horizontal cell to hyperpolarize, thus leading to an increase in the current flowing through both the hemichannels and the glutamate-gated channels [7], [9]. This current flow produces a voltage drop along the high resistance path of the synaptic cleft, thereby shifting the Ca^2+^-current in the cones to more negative potentials and enhances glutamate release from the cone terminal.

An alternative view of the mechanism that mediates feedback in the distal retina was put forth by Hirasawa and Kaneko [10]. They found that the feedback-induced shift of the Ca^2+^-current in cones was inhibited when extracellular proton fluctuations were stabilized by a high concentration of the pH buffer HEPES; the high concentration of HEPES also reduced light-induced surround effects in bipolar cells. These data together with evidence that increasing extracellular pH shifts the L-type Ca^2+^-current toward negative potentials [11] led them to conclude that protons regulate the horizontal cell-to-cone feedback pathway (Fig. 1B).

In the present study, we performed a series of electrophysiological experiments designed to test the pH-mediated feedback mechanism and to determine whether the effects of extracellular pH buffering can also be evaluated in terms of the ephaptic feedback hypothesis. A major feature of these experiments is the use of a series of pharmacological agents that change the intracellular pH or the extracellular pH buffering. Our experimental findings suggest that artificial pH buffers, on which several key arguments supporting the pH hypothesis are based, induce intracellular acidification in addition to clamping the extracellular pH. Because hemichannels are inhibited by intracellular acidification, these experiments do not seem to discriminate between a pH-mediated or a hemichannel-mediated mechanism. Independent experiments to further test the pH hypothesis failed to generate support for the pH-mediated mechanism. Moreover, the experimental results concerning the endogenous pH buffering system, and the computational analysis, are in line with an ephaptic feedback pathway that operates through both hemichannels and glutamate-gated channels.

## Results

### Effect of pH buffering on feedback responses in cones and horizontal cells

Several studies have shown that HEPES, a rapidly acting pH buffer, significantly reduces the feedback response [10], [12]–[14]. The reduction of feedback approximately followed the buffer capacity of the various pH buffers added to the Ringer's solutions [14] consistent with the idea that extracellular pH changes mediate feedback. Because Davenport et [14] measured surround responses of ganglion cells and roll-back responses in horizontal cells, which are indirect measures of cone feedback, we repeated their experiments on goldfish retina in order to compare the effect of HEPES on feedback responses measured directly in cones and indirectly in horizontal cells.

**Figure 2:**
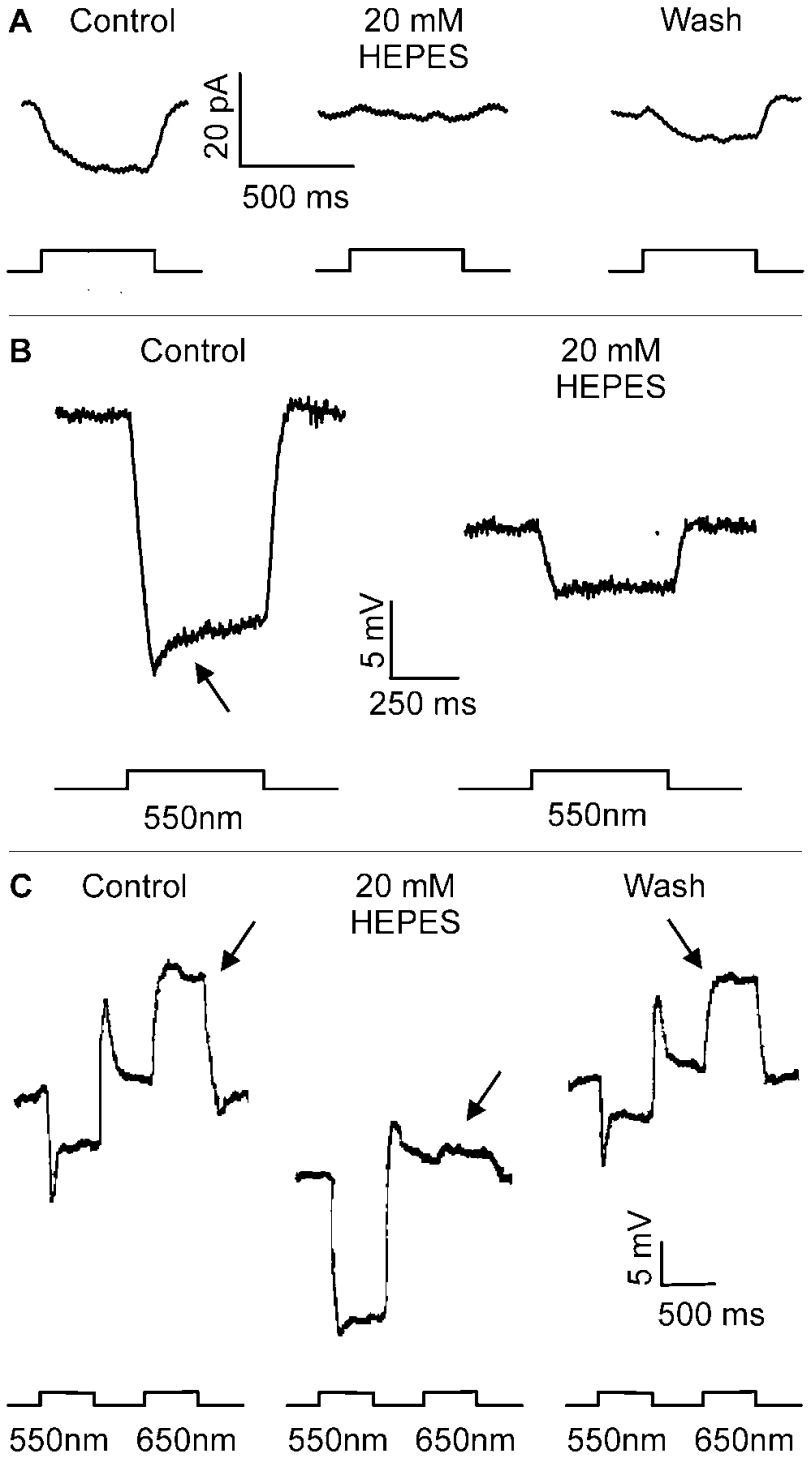
HEPES reduces feedback-mediated responses in the cone/horizontal cell network in the isolated retina of goldfish. *A*, Feedback responses elicited by a 500 ms full-field (3000 µm) stimulus (I = 0 log) delivered in the presence of a 20 µm spot of light (I = 0 log) centered on a cone voltage-clamped at −45 mV. Current responses are shown in control (left), in the presence of 20 mM HEPES (middle), and after washout (right). HEPES reduces the amplitude of the feedback-mediated responses. *B*, Responses of a monophasic horizontal cell to 550 nm light (I = 1 log) in control (left), and after application of 20 mM HEPES (right). HEPES hyperpolarizes horizontal cells and strongly reduces the light-induced hyperpolarization and the feedback-induced roll-back response (arrow). *C*, Responses of a biphasic horizontal cell to light of 550 nm (I = 1 log) and 650 nm (I = 1 log) in control (left), after application of 20 mM HEPES (middle), and after washout (right). HEPES reduces the feedback-induced depolarizing response due to deep red light stimulation (arrows).

Feedback responses were elicited by a 500 ms 3000 µm stimulus (I = 0 log), which was delivered in the presence of a 20 µm spot of light (I = 0 log) centered on a cone voltage-clamped at −45 mV. With this stimulus paradigm, 3000 µm stimulation induced an inward current in cones which has been identified as a pure feedback-induced modulation of the Ca^2+^-current [4], [10], [15]. Secondary to this increase in Ca^2+^-current, a delayed outward Ca^2+^-dependent Cl–current can appear [15]. In this paper we focus on the feedback induced inward current. Fig. 2A shows that the feedback-induced inward current is greatly reduced in the presence of 20 mM HEPES (middle panel); the response reappeared immediately after washout (right panel). In the 10 cones tested, 20 mM HEPES reduced the feedback-mediated response by 79.8±7.1% (p≤0.05).

Feedback signals are also imposed on the light response of horizontal cells. Fig. 2B shows the full-field light response of a monophasic horizontal cell with the feedback-mediated secondary depolarization (roll-back) indicated by the arrow. Application of 20 mM HEPES hyperpolarized the horizontal cell, reduced its light response amplitude, and almost completely eliminated the roll-back. In the 5 cells tested, the horizontal cell membrane potential hyperpolarized by 9.7±1.0 mV (p≤0.05), the light response amplitude was reduced by 48.0±8.2% (p≤0.05), and the roll-back response was reduced by 95.2±4.8% (n = 5; p≤0.05). Biphasic horizontal cells hyperpolarized in the presence of 20 mM HEPES, and the roll-back from the response peak was diminished (Fig. 2C). Moreover, the feedback-mediated depolarizing response to the 3000 µm spot of red light stimuli present in control conditions was greatly reduced and reappeared after washout (arrows in Fig. 2C). On average, the reduction of the feedback-induced depolarizing response was 90.3±5.0% (p≤0.05) (n = 3).

### The effect of pH buffer strength on the feedback signal

The fact that increasing the pH buffering capacity of the extracellular milieu with HEPES suppressed the light-induced feedback signal raises the question of whether the feedback or the feedforward signal was affected. Any decrease in the feedforward signal will obviously lead to a decrease in the light-induced feedback-signal, because the feedback pathway will receive less input. Because our findings indicate that HEPES reduced the light response of horizontal cells (Fig. 2B), the data of Fig. 2 do not conclusively show that the feedback signal has been reduced by increasing the pH buffer capacity. To address this problem, one has to bypass changes in the light response amplitude of horizontal cells that might have been induced by the pharmacological manipulations. A voltage clamp approach is not feasible since horizontal cells are strongly electrically coupled and application of dopamine leads only to a reduction and not to a complete loss of coupling [16]–[18]. Therefore, we have chosen to globally polarize horizontal cells by modulating their glutamate receptors pharmacologically.

**Figure 3:**
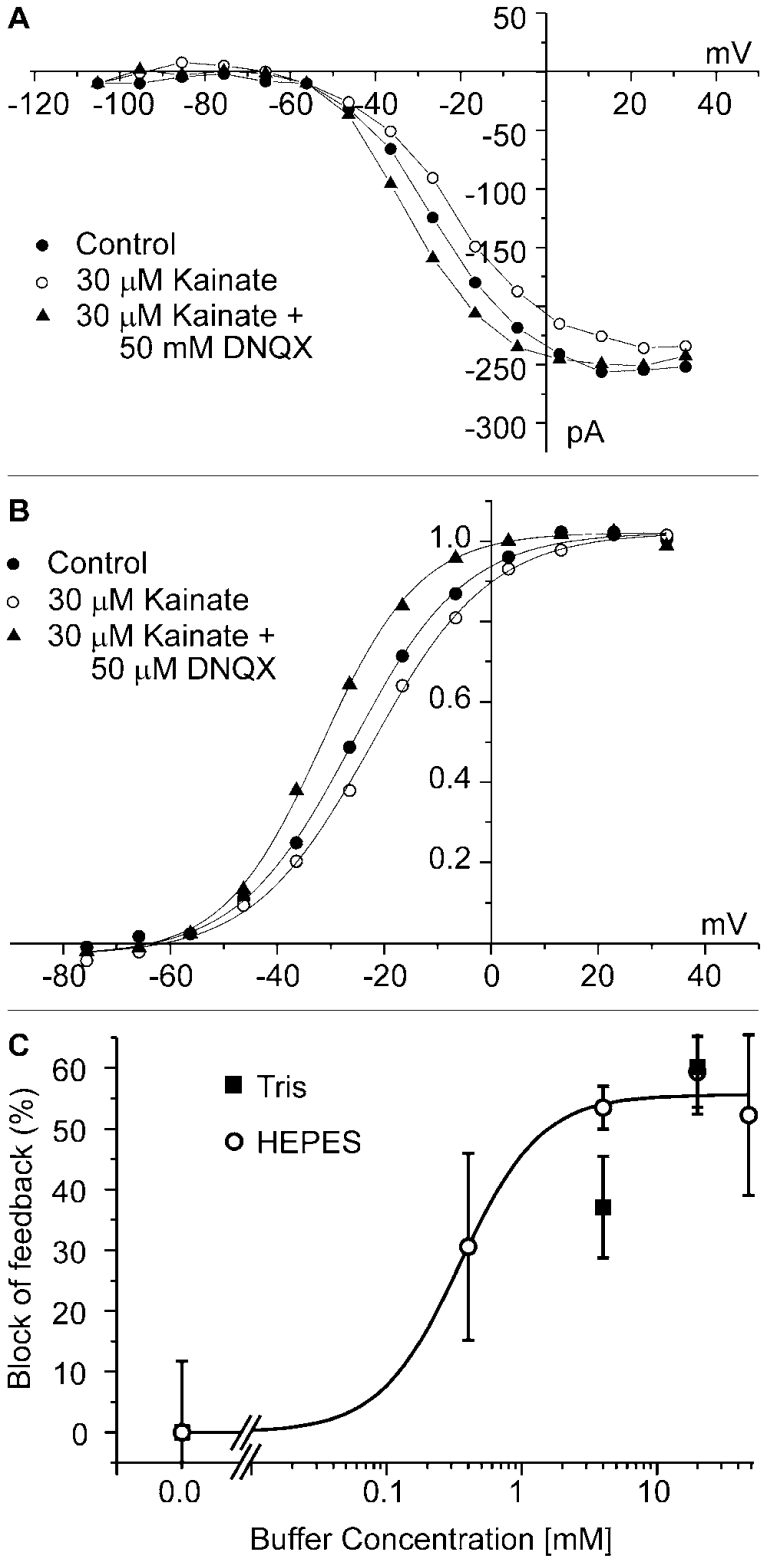
HEPES does affect the kainate-DNQX induced shift of the Ca^2+^-current in a dose-dependent manner in the isolated retina of goldfish. *A*, Ca^2+^-current of the cone in control bicarbonate-based Ringer's solution (filled circles), after depolarization of horizontal cells with 30 µM kainate (open circles), and after subsequent hyperpolarization of horizontal cells by the addition of 50 µM DNQX (filled triangles). *B*, Boltzmann fits through the activation functions of the Ca^2+^-current from the same cell shown in (A). 30 µM kainate shifts the Ca^2+^-current to more positive potentials, whereas a subsequent application of 50 µM DNQX shifts the Ca^2+^-current to more negative potentials. The difference of the half activation in kainate and in DNQX is 9.3±1.1 mV (n = 7) and is a measure for the feedback strength independent of light responses. *C*, Inhibition of maximal shift of activations of the Ca^2+^-current in control, and in the presence of various concentrations of HEPES (open symbols) or Tris (closed symbols). The solid line represents a fitted Hill equation though the HEPES data points. Compared to Tris, HEPES is a more potent inhibitor of feedback.

Application of 30 µM kainate, an agonist of AMPA receptors, will strongly depolarize horizontal cells, and application of 50 µM DNQX, a blocker of AMPA receptors, will hyperpolarize horizontal cells. Neither drug affects cones directly as is evident from the following findings. The holding current of cones clamped at −60 mV is: −100.5±19.0 pA (n = 6) in control, −93.5±16.6 pA (n = 7) in kainate and −94.4±18.1 pA (n = 7) in kainite+DNQX. These values do not differ significantly from each other. The application of DNQX following kainate leads to a reversible hyperpolarization of the horizontal cell membrane potential of 52.7±1.4 mV (n = 5). Correlating this membrane potential change with the shift of the Ca^2+^-current in cones gives an estimate of the strength of feedback. 3A shows the voltage-activated Ca^2+^-currents of a cone in control Ringer's solution (filled circles), after the horizontal cells have been maximally depolarized by the glutamate agonist 30 µM kainate (open circles), and after they have been hyperpolarized by the addition of 50 µM DNQX (filled triangles). In the 7 cells tested, the maximal amplitude of the Ca^2+^-current did not significantly change in the presence of either kainate or DNQX compared to control conditions. Fig. 3B shows the Boltzmann fits to activation curves of the same Ca^2+^-currents shown in Fig. 3A. With respect to control (filled circles), 30 µM kainate shifted the Ca^2+^-current to more positive potentials (open circles), whereas the curve shifted to more negative potentials with the addition of 50 µM DNQX (filled triangles). The pharmacologically-induced shift of the Ca^2+^-current, i.e. the difference between the half maximal potential recorded in 50 µM DNQX and in 30 µM kainate, was 9.3±1.1 mV (n = 7). This is the maximal inducible shift of the Ca^2+^-current.

Using the aforementioned procedure, the effects of a concentration series of HEPES was tested. The buffer capacity of HEPES in each solution is given in the Materials and Methods section. While 48 mM HEPES blocks proton-mediated processes in cone and bipolar synapses by 90%, 3 mM HEPES does not inhibit these processes [19]. This suggests that the concentration for half maximal block is well above 3 mM. Fig. 3C shows the dose response curve for HEPES inhibition of the maximal inducible shift of the Ca^2+^-current as described above. HEPES maximally inhibits feedback by about 60% with a K_d_ of about 350 µM. The kainate/DNQX induced hyperpolarization of horizontal cells was not affected in the various HEPES solutions; control: 52.7±1.4 mV (n = 5), 4 mM HEPES: 55.0±4.3 mV (n = 5), 20 mM HEPES: 54.9±4.7 mV (n = 5), 48 mM HEPES: 54.3±5.6 mV (n = 6). Fig. 3C also gives data points for Tris. 4 mM Tris inhibits feedback by about half showing that this buffer is less effective than HEPES at blocking feedback. This was surprising because Tris and HEPES have nearly equal buffer capacities (see Materials and Methods) in our recording conditions (pH 7.8; 4 mM HEPES: 2.0 mM versus 4 mM Tris: 2.1 mM). A possible source of this difference could be a direct effect of HEPES on hemichannels, which has been suggested to play a role in negative feedback from horizontal cells to cones [7]. In contrast to Tris, all aminosulfonate-based buffers, such as HEPES, can inhibit connexin hemichannels [20], [21].

**Figure 4:**
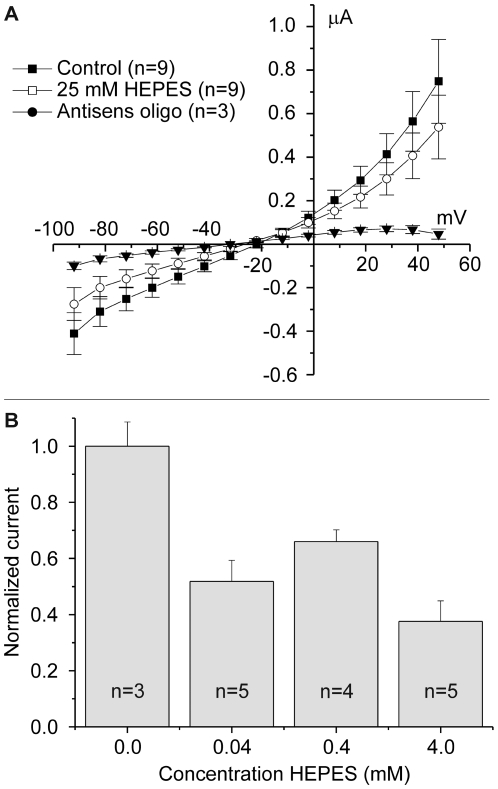
HEPES inhibits Cx55.5 hemichannel-mediated currents in Xenopus oocytes. *A*, Mean currents of 9 oocytes are plotted as function of potential. The control current (filled squares) is inhibited by application 25 mM HEPES (open circles). No current is present in antisense injected oocytes (filled triangles). Error bars =  ±sem. *B*. The mean reduction in Cx55.5 hemichannel current at potentials between −55 and −35 mV. At a concentration of 4 mM HEPES, the hemichannel mediated current decreased to 37.9±0.07% (n = 7; p<0.001). All tested concentrations of HEPES induced a significant reduction of the Cx55.5 hemichannel current (40 µM HEPES: p = 0.0065, n = 5; 0.4 mM HEPES: p = 0.0118, n = 4; 4.0 mM HEPES: p = 0.00165, n = 5).

To test whether HEPES affected the horizontal cell hemichannels directly, we expressed Cx55.5 [22] in oocytes and measured hemichannel-mediated currents. Fig. 4A depicts the hemichannel currents recorded in control conditions (filled squares) and after addition of 25 mM HEPES (open circles). The inhibitory effect of HEPES on Cx55.5 hemichannels was seen in each of nine oocytes tested. Antisense injections did not lead to the observed currents (filled triangles). The effect of various dosages of HEPES on Cx55.5 hemichannel currents was determined. Oocytes were held at −70 mV and stepped for 10 sec to potentials ranging from −100 mV to+10 mV. Sustained currents at the end of the step were measured. The mean reduction in current was determined at potentials between −40 and −20 mV. All three concentrations led to a significant reduction of the hemichannel current, indicating that hemichannels are very sensitive to HEPES (Fig. 4B). At a concentration of 4 mM HEPES, the hemichannel mediated current decreased to 37.9±0.07% (n = 7; p<0.001). The experimental results presented so far indicate that application of artificial pH buffers leads to a partial inhibition of feedback, that the strength of inhibition of feedback does not strictly follow the pH buffer capacity, and that HEPES affects hemichannels directly.

**Figure 5:**
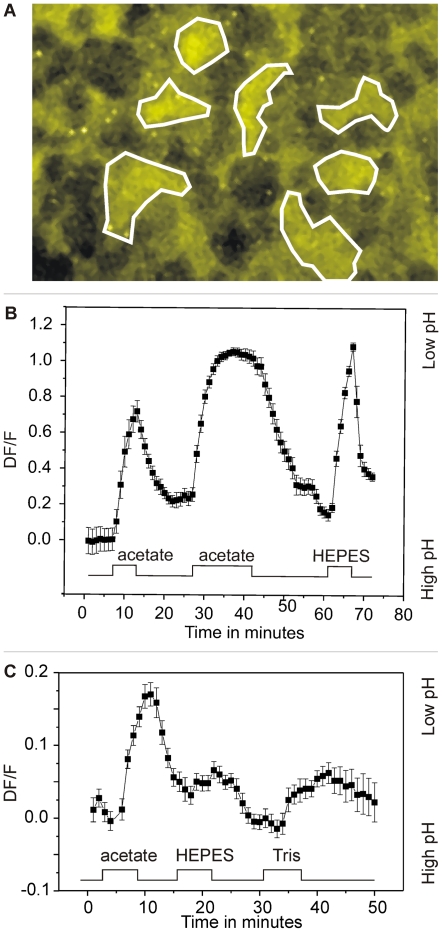
HEPES and Tris lead to intracellular acidification in the isolated retina of goldfish. *A*, BCECF fluorescence of horizontal cells in the flat mounted retina in control conditions. Horizontal cells stain yellow. *B and C*, ΔF/F as function of time. Data points are the average of 7 cells (regions of interest) and error bars represent sem. Increase in fluorescence signal indicates acidification. Complete exchange of solution in recording chamber took about 2 minutes. Frames were sampled every 2 minutes. Application of 25 mM acetate, 20 mM HEPES and 20 mM TRIS all lead to a reduction of intracellular pH.

Next, we tested the implicit assumption that artificial buffers like HEPES and Tris buffer only affects the extracellular pH without affecting the intracellular pH. Using a two-photon microscope and the pH dye BCECF, we measured changes in cone and horizontal cell intracellular pH in the isolated retina. In our experimental conditions, an increase in fluorescence indicates a more acidic environment (see methods). Fig. 5A shows the fluorescence at the level of the horizontal cells. The white lines indicate the regions of interest and the black circular structures indicate the nuclei of horizontal cells. BECEF is known not to enter the nuclei [23]. Acetate in its protonated form readily traverses the cell membrane, dissociates to release protons, and leads to intracellular acidification. For example, in catfish horizontal cells, application of 25 mM acetate led to an intracellular decrease in pH of about 0.6 units [24]. Fig. 5B shows the increase in fluorescence in horizontal cells after application of 25 mM acetate, confirming that acetate indeed leads to a decrease in the intracellular pH. This validates the technique. When 20 mM HEPES was applied, a similar decrease in pH was observed. This was found in all 5 retinas tested, both at the level of the horizontal cells and at the level of the synaptic terminals of cones (data not shown). Intracellular acidification could also be observed in all three retinas tested with 4 mM HEPES, although the effect was smaller. Fig. 5C shows that 20 mM Tris leads to an increase in fluorescence in horizontal cells and thus to a decrease in pH, similar to acetate and HEPES. This was found in all three retinas tested. These results indicate that, apart from buffering pH in the extracellular compartment, artificial buffers such as HEPES and Tris lead to a decrease in intracellular pH in both horizontal cells and cones, leaving open the question as to which of these two effects underlies the inhibition of feedback.

**Figure 6:**
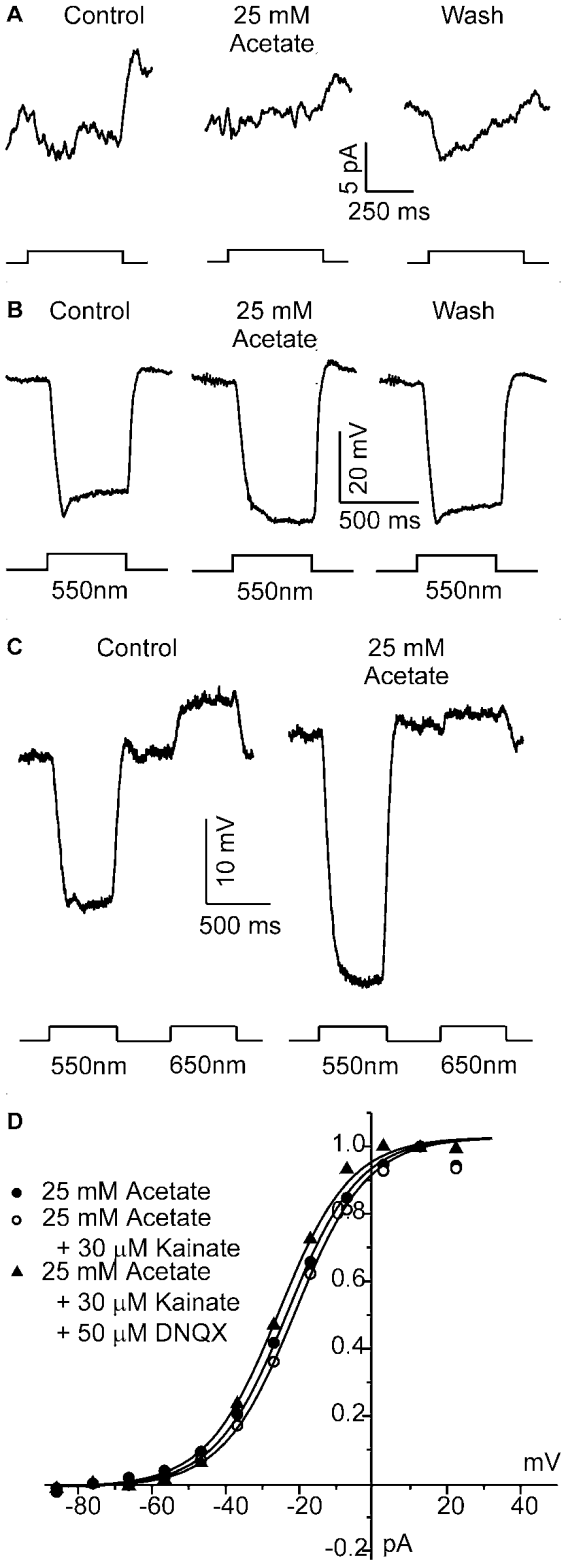
Intracellular acidification reduces feedback-mediated responses without hyperpolarizing horizontal cells in the isolated retina of goldfish. *A*, Feedback-responses elicited by a 500 ms full-field stimulus (I = 0 log) delivered in the presence of a 20 µm spot of light (I = 0 log) centered on a cone clamped at −38 mV. Current responses are shown in control (left), after application of 25 mM acetate (middle), and after washout (right). Acetate reduces feedback-induced inward currents in cones. *B*, Responses of a monophasic horizontal cell to 550 nm light (I = 0 log), in control, after application of 25 mM acetate (middle), and after washout (right). Intracellular acidification blocks the feedback-induced roll-back response without affecting the resting membrane potential of the horizontal cells. *C*, Responses of a biphasic horizontal cell to light of 550 nm (I = 1 log) and 600 nm (I = 1 log) in control (left), and after application of acetate (right). Acetate reduces the feedback-induced depolarization. *D*, Boltzmann fits through the activation function of the Ca^2+^-current of a cone in 25 mM acetate. 30 µM kainate shifts the Ca^2+^-current to more positive potentials, whereas a subsequent application of 50 µM DNQX shifts the Ca^2+^-current to more negative potentials.

Next, we studied the effect of intracellular acidification on feedback responses in the absence of a pronounced change in pH buffer capacity. Acetate leads to intracellular acidification, but it is also a weak pH buffer. In fact, the pH buffer capacity of 25 mM acetate is about 240 times less than that of 25 mM HEPES. Thus, if the pH buffer component is the most prominent factor affecting feedback, acetate should be ineffective in inhibiting feedback. On the other hand, if intracellular acidification is the basis of feedback inhibition, acetate should be very effective. Fig. 6A shows that addition of 25 mM acetate to the Ringer's solution strongly attenuated the feedback-mediated responses in cones (mean 71.0±7.7%; n = 6; p<0.05). Although the cell depicted here recovered the feedback response after washout, in most cells recovery was not complete. Acetate also suppressed the roll-back response in horizontal cells (Fig. 6B). In the 10 cells tested, acetate reduced the roll-back by 97.1±2.8% (p<0.05), without a significant effect on either the dark resting membrane potential (−3.2±2.7 mV; p>0.05) or the amplitude of the light response (−8.3±10.2%; p>0.05). The feedback-induced response of a biphasic horizontal cell to red light stimulation was also greatly reduced (by 76.5±10.6%; n = 5; p<0.05) when acetate was applied to the Ringer's solution (Fig. 6C).

To provide a more quantitative assessment of the degree to which feedback has been inhibited, the kainate-DNQX induced shift of the Ca^2+^-current was measured in the presence of 25 mM acetate. Fig. 6D shows the activation function of the cone Ca^2+^-current in acetate (solid circles), after application of 30 µM kainate (open circles), and after subsequent application of 50 µM DNQX (solid triangles). As in control, kainate shifted the Ca^2+^-current to more positive potentials and DNQX to more negative potentials. However, the displacement was significantly (p<0.05) smaller in acetate (3.4±0.2 mV; n = 5) than in control (9.3±1.1 mV; n = 7), an indication that intracellular acidification led to inhibition of feedback.

The results presented above suggest that application of artificial buffer systems to block pH changes in the synaptic cleft acts to inhibit feedback and decrease the intracellular pH. Manipulations that do not affect the extracellular pH buffering, but only decrease the intracellular pH, lead to a similar inhibition of feedback. Next, we tested the pH-hypothesis independent of the use of artificial buffers by modifying the endogenous buffer system.

### Testing the pH-hypothesis independent of artificial buffer systems

**Figure 7:**
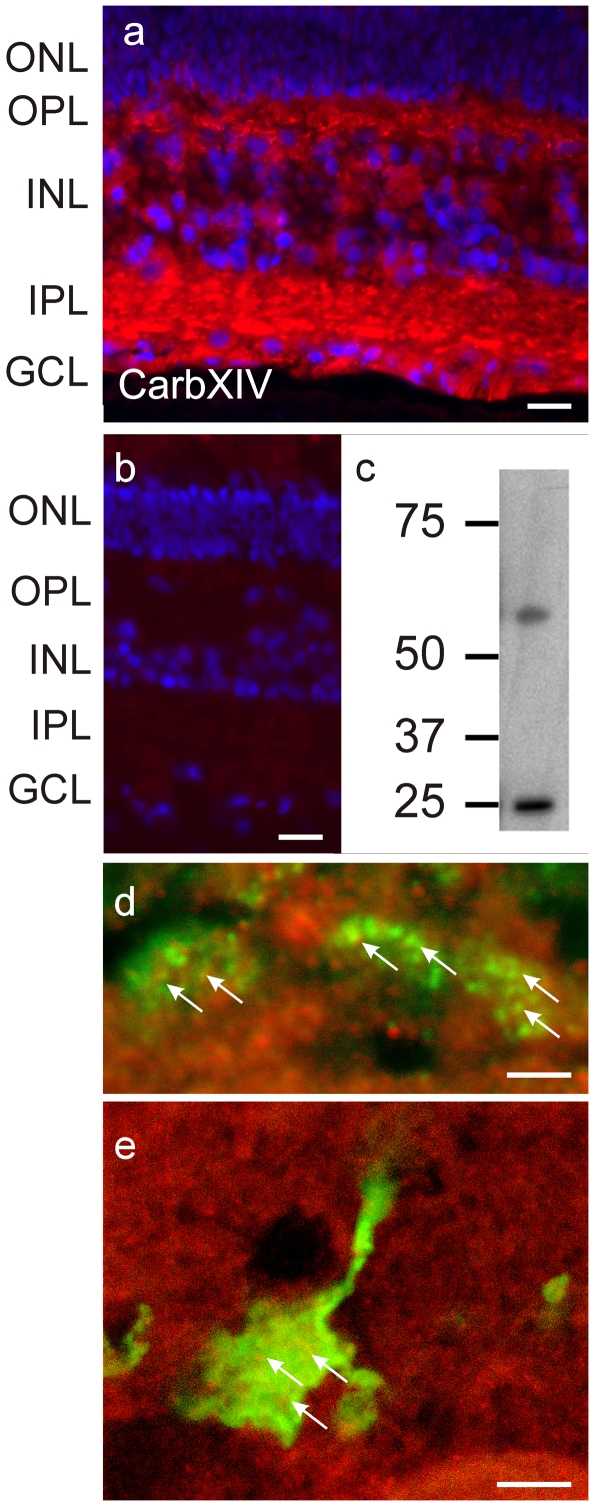
Localization of extracellular carbonic anhydrase XIV in the goldfish retina. *A*, Distribution of the extracellular carbonic anhydrase in the goldfish retina. Immunostaining by an antibody raised against the mouse extracellular carbonic anhydrase XIV was found throughout the goldfish retina. Extensive labeling (red) was found throughout the OPL, suggesting a role for extracellular carbonic anhydrase in synaptic transmission between cones and horizontal cells. The blue label is due to the nuclear marker DAPI. ONL: outer nuclear layer, OPL: outer plexiform layer, INL: Inner nuclear layer, IPL: inner plexiform layer. *B*, No label remains after pre-adsorption of the primary antibody with the immunizing recombinant carbonic anhydrase XIV. Scale bars in A and B = 20 µm. *C*, Western blot of membrane fractions of goldfish retina shows a band at about 57 kDA and a band at 25 kDa. The 25 kDa band represent a proteolytically nicked form of carbonic anhydrase (A. Waheed and W. Sly, personal communication). *D*, Distribution of carbonic anhydrase (red) relative to GluR2 (green) in the cone synaptic terminal. GluR2 labels invaginating dendrites of horizontal cells in cone terminals. Yellow (arrows) indicates a close association between carbonic anhydrase and GluR2, showing synaptic localization of carbonic anhydrase. Scale bar = 2 µm. *E*, Distribution of carbonic anhydrase (red) and PKC-α (green) in the bipolar cell synaptic terminal. Yellow (arrows) indicates a close association between carbonic anhydrase and PKC, showing synaptic localization of carbonic anhydrase. Scale bar = 5 µm.

Bicarbonate is the endogenous pH buffer system. In the absence of the enzyme carbonic anhydrase, bicarbonate is known to act on an extremely slow time scale [25]; i.e. it will not buffer the fast changes in proton concentration induced by neuronal activity. However, biological systems express the enzyme carbonic anhydrase, which is one of the fastest regulatory enzymes known [26]. The enzyme catalyzes the reversible hydration of CO_2_, thereby giving the bicarbonate-based pH buffer system a high buffer capacity (see Materials and Methods). To determine whether extracellular carbonic anhydrase is present in the outer retina, we examined the distribution of the extracellular membrane-bound isoform of carbonic anhydrase (type XIV) in the goldfish retina using an antibody raised against the mouse carbonic anhydrase XIV [27], [28]. Immunoreactivity was seen throughout the neural retina (Fig. 7A). The outer and inner plexiform layers showed the highest intensity of labeling, consistent with previous reports indicating that the enzyme is most prominent on the radial glia whose processes interdigitate with every retinal neuron. Fig. 7B shows the pre-adsorption control and Fig. 7C the Western blots, illustrating the specificity of the antibody in goldfish retina. The band at about 57 kDa is close to the 54 kDa band seen in Western blots of the mouse retina [27]. The band around 23 kDa represents proteolytically nicked carbonic anhydrase XIV (A. Waheed and W. Sly: personal communication). The presence of extracellular carbonic anhydrase XIV suggests that a high capacity pH buffer system is present near the location where synaptic transmission between cones and horizontal cells takes place. To determine whether carbonic anhydrase was present within the synaptic complex of both cones and bipolar cells, we performed double labeling experiments with GluR2, a marker for horizontal cell processes invaginating in the cone synaptic terminal [29] and with PKC-α, a bipolar cell marker. Fig. 7D shows the outer retina double labeled with antibodies against GluR2 (green) and carbonic anhydrase type XIV (red). The GluR2 labeling is covered by yellow dots suggesting a close association of carbonic anhydrase type XIV and the cone synaptic terminal. Fig. 7E shows a bipolar cell synaptic terminal labeled with an antibody against PKC-α (green). The red label indicates carbonic anhydrase type XIV immunoreactivity. Yellow dots appear on the PKC-α label, suggesting a close association of carbonic anhydrase type XIV and the bipolar cell synaptic terminal.

**Figure 8:**
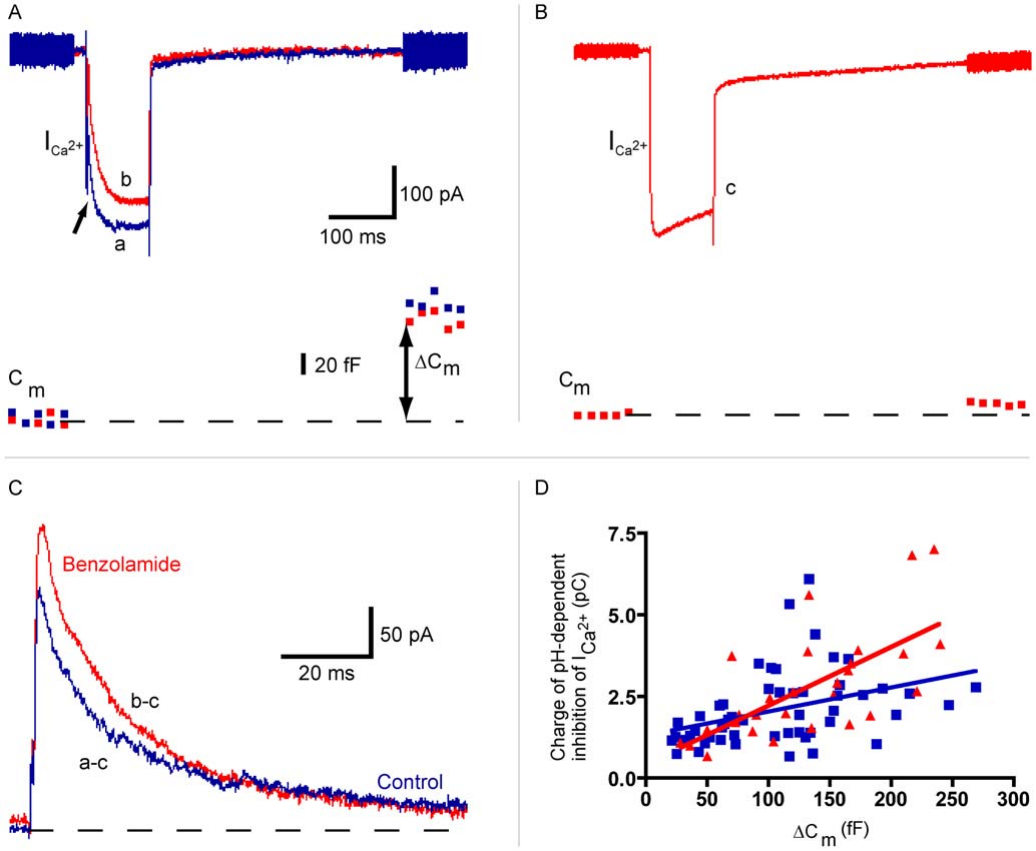
Benzolamide enhances pH-dependent inhibition of L-type Ca^2+^ currents in isolated goldfish Mb bipolar cell terminals. A, Bath application of 100 µM benzolamide and 100 µM picrotoxin enhances the “pH-nose” inhibition of L-type Ca^2+^ currents elicited by 100 ms steps from −60 mV to −10 mV. This inhibition is mediated by the exocytosis of protons co-packaged with glutamate. Upper traces: Individual voltage-clamp traces from two different isolated terminals in the same slice. In the continuous presence of picrotoxin (100 µM; a: blue trace), a 100 ms step to −10 mV produces an inward Ca^2+^ current of nearly 400 pA that is inhibited by a rapidly decaying pH-nose (indicated by arrow) immediately following the onset of depolarization. Addition of benzolamide (100 µM; b: red trace) enhances the amplitude and area of the pH-nose. Voltage-clamp sine waves before and after the depolarizing step allow for the measurement of membrane capacitance change (ΔC_m_; exocytosis). Lower traces: Measurements of ΔC_m_ corresponding to the traces shown above. The ΔC_m_ in control is 158 fF (blue trace) and it is 132 fF (red trace) in the presence of 100 µM benzolamide. B, The pH-nose is absent from a pure Ca^2+^ current, elicited by a 100 ms step to −10 mV following nearly complete run-down of exocytosis. Upper traces: Depolarization of an isolated terminal to 10 mV for 100 ms produces an inward Ca^2+^ current of nearly 400 pA (c: red trace). Lower trace: The corresponding capacitance trace shows that exocytosis is almost completely absent. C, The exact charge transfer for each pH-dependent inhibition of I_Ca2+_ in (A) was calculated by scaling the original traces (traces a,b) to match the amplitude of the Ca^2+^ current in (B), subtracting the pure Ca^2+^ current trace (trace c) from the Ca^2+^ current traces with pH noses (a,b), and integrating the area under the difference curve during the 100 ms depolarization to −10 mV. The pH-nose charge in the presence of 100 µM benzolamide (b–c) is 3.878 pC, and the pH-nose in control (a–c) is 2.854 pC. D, Bath application of 100 µM benzolamide (red triangles; n = 29, 6 cells; control is blue squares; n = 53, 8 cells) significantly increases the positive slope of the relationship between the size of the charge of pH-dependent inhibition of I_Ca2+_ and ΔC_m_. The slope of the linear regression for 100 µM benzolamide (red line; 0.018+/−0.004) was significantly non-zero (p<0.0001, r^2^ = 0.45) and significantly larger than the slope for control (p = 0.017). The slope for control (blue line; 0.007+/−0.002) was also significantly non-zero (p = 0.004, r^2^ = 0.15).

In brain it has been shown that application of benzolamide, a membrane impermeant inhibitor of carbonic anhydrase, leads to about three times larger changes in extracellular pH [reviewed in: 30]. If protons are the feedback neurotransmitter, one would expect feedback-mediated responses to increase after application of benzolamide. To determine whether application of benzolamide resulted in larger extracellular pH changes, we first used the protocol previously described by Palmer [19]. They found that the Ca^2+^-current (I_Ca_) in bipolar cell terminals was transiently inhibited by the exocytosis of protons co-released with glutamate, resulting in a so-called “pH-nose” on the Ca^2+^-current (Fig. 8A; a, upper blue trace, arrow). The pH-nose has also been identified in cones [31] and reflects pH changes in the synaptic cleft near the synaptically localized Ca^2+^-channels.

Ca^2+^-currents were elicited by a 100 ms depolarization from −60 to −10 mV in the presence of 100 µM picrotoxin to avoid GABAergic feedback [32]. During activation of the Ca^2+^-current, exocytosis of glutamate occurred, as evident from the change in whole-cell capacitance (Fig. 8A; lower blue trace; von Gersdorff et al., 1998). The release of vesicular protons induced a pH-dependent inhibition of Ca^2+^ current (“pH nose”). Following run-down of glutamate release, it is possible to record a pure Ca^2+^ current without a pH nose (Fig. 8B; c, upper red trace). Subtracting the control Ca^2+^-current (c), elicited following complete, or nearly complete, run-down of exocytosis, from the Ca^2+^ current with the pH nose (7A; a or b) yields the pure “pH nose” current (Fig. 8C; a–c: control trace, blue; b–c: 100 µM benzolamide, red). However, note that we first scaled the control Ca^2+^-current (c) to the peak Ca^2+^ current with the pH nose (Fig. 8A; a or b) before we performed this subtraction of Ca^2+^ currents, to avoid a possible effect of Ca^2+^ current rundown.

When carbonic anhydrase is inhibited by 100 µM benzolamide, the pH-nose in bipolar cell terminals becomes on average significantly larger (control - blue trace; benzolamide - red trace). To compare the size of the pH-nose quantitatively, it was isolated from the Ca^2+^-current (Fig. 8C) and integrated to obtain the total charge transfer in pC units. These values were plotted as a function of ΔC_m_, the capacitance change, an index of the magnitude of vesicular exocytosis and glutamate release [33]. Bath application of 100 µM benzolamide (Fig. 8D; red triangles; n = 29, 6 cells) in the continuous presence of 100 µM picrotoxin (Fig. 8D; blue squares; n = 53, 8 cells) significantly increases the positive slope of the relationship between the charge of pH-dependent inhibition of I_Ca2+_ and ΔC_m_. The slope of the linear regression for 100 µM benzolamide (Fig. 8D; red line; 0.018+/−0.004) was significantly greater than zero (p<0.0001, r^2^ = 0.45) and significantly larger than the slope for control alone (Fig. 8D; blue line; 0.007+/−0.002, p = 0.017). This indicates that inhibition of carbonic anhydrase by benzolamide reduces the pH buffering and thus allows for larger changes in synaptic cleft pH, which in turn results in increased inhibition of the Ca^2+^ current. Recordings in which a lower concentration of benzolamide (10 µM) was used (n = 18, 8 cells, slope = 0.005±0.002) showed no significant difference (p = 0.51) in the slope of the relationship between the charge of pH-dependent inhibition of I_Ca2+_ and ΔC_m_ compared to control recordings (Fig. 8D; blue line; n = 11, 7 cells, slope = 0.007±0.002). To determine if application of 100 µM benzolamide affected steady-state Ca^2+^-currents during 100 ms depolarizing steps, the mean Ca^2+^-current amplitude for the final 75 ms of each step was calculated for all Ca^2+^-current traces. There was no significant difference between the mean Ca^2+^-current amplitudes for both conditions (control = −427.4±14.5 pA, n = 32; benzolamide = −463.5±23.1 pA, n = 24; p = 0.17).

These experiments show that benzolamide enhances the pH-nose inhibition of Ca^2+^ current in bipolar cell synaptic terminals. To test whether a similar effect could be found in cones, we recorded the pH-nose of the cone Ca^2+^-current, with and without 100 µM benzolamide in the bath. Because we cannot record from the cone synaptic terminal directly, as can be done for bipolar cells, we were unable to combine these measurements with capacitance measurements. Nevertheless, on average, the “pH-nose” of the Ca^2+^-current in cones increased by 44±19% (n = 6; p = 0.03981) after application of benzolamide, suggesting that pH-buffering in the synaptic cleft of cones was reduced as well.

**Figure 9:**
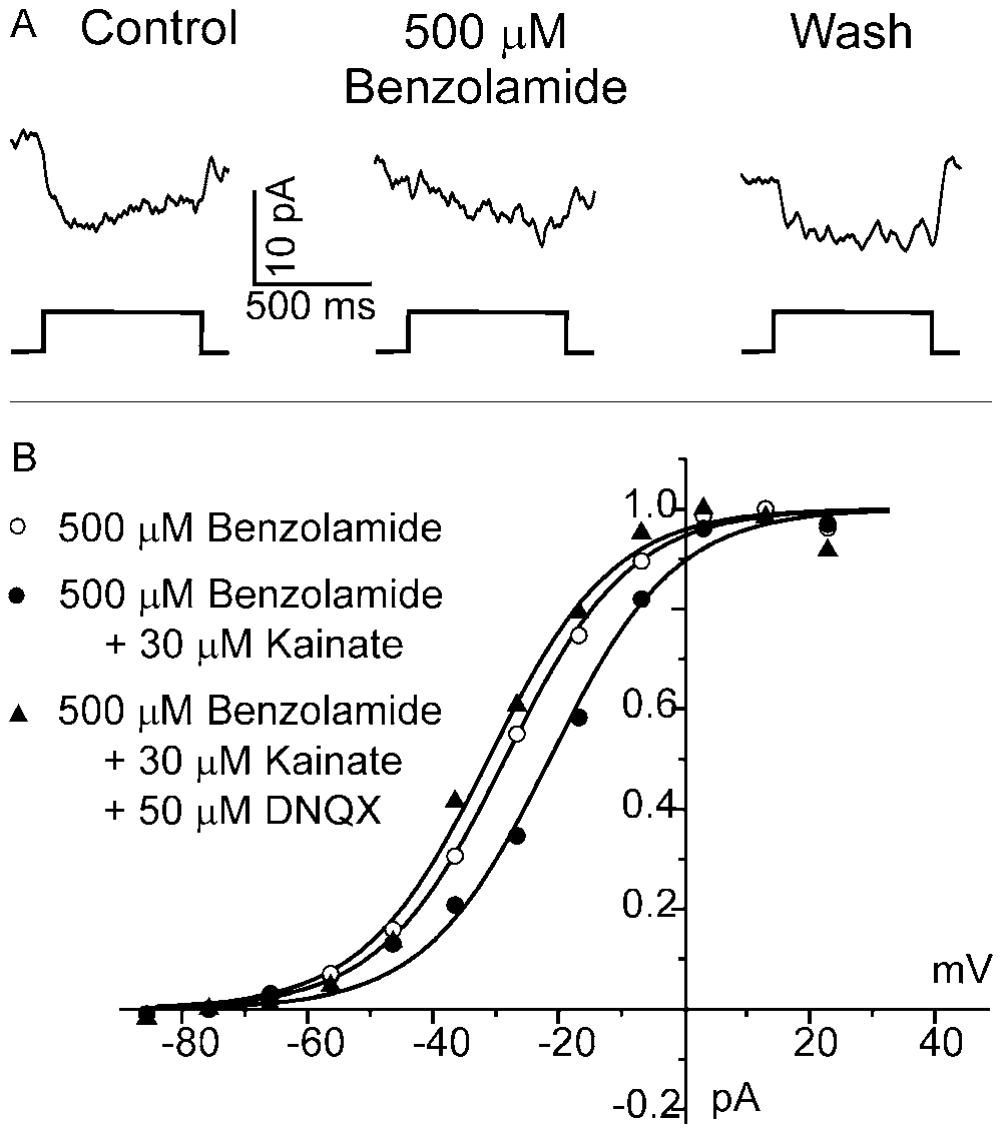
Benzolamide does not increase the feedback-induced inward currents in cones in the isolated goldfish retina. *A*, Feedback-responses elicited by a 500 ms full-field stimulus (I = 0 log) delivered in the presence of a 20 µm spot of light (I = 0 log) centered on a cone clamped at −43 mV. Current responses are shown in control (top), after application of 500 µM benzolemide (middle), and after washout (bottom). *B*. Feedback induced shift of the Ca-current of a cone in 100 µm benzolamide. 30 µM kainate shifts the Ca^2+^-current to more positive potentials, whereas a subsequent application of 50 µM DNQX shifts the Ca^2+^-current to more negative potentials. The difference of the half activation in kainate and in DNQX is 7.0±1.6 mV (n = 6).

Next, the effect of benzolamide on feedback from horizontal cells to cones was determined. Fig. 9A shows that 500 µM benzolamide reversibly reduced the light-induced feedback currents in cones. The mean benzolamide-induced reduction in feedback was 59.0±11.0% (n = 6; p<0.05). Benzolamide application had no effect on either the Ca^2+^-current amplitude (control: 218±53 pA; benzolamide: 226±50 pA; n = 6; p>0.05), the cone light response (control: −11.2±2.0 mV; benzolamide: −12±2.3 mV; n = 5; p>0.05), or the resting membrane potential of the cones (control: −34±1.2 mV; benzolamide: −34.1±0.9 mV; n = 5; p>0.05). Contrary to what is predicted by the pH hypothesis, benzolamide does not lead to an increase in feedback responses. To circumvent the possibility that benzolamide induced changes in light responsiveness of cones and horizontal cells, we measured the shift of the Ca^2+^-current due to polarization of horizontal cells by using the kainate/DNQX protocol. Fig. 9B shows the Ca^2+^-current of the cone in the presence of benzolamide (open circles), after addition of 30 µM kainate (solid circles) and in the presence of 50 µM DNQX (triangles). In 500 µM benzolamide, this shift was 7.0±1.6 mV, not significantly different from the shift obtained in the absence of benzolamide (9.5±1.1 mV; n = 7; p>0.05). In the presence of benzolamide, the polarization of horizontal cells due to kainate and DNQX was −64.9±4.4 mV, a slight increase compared to the polarization by kainate and DNQX in the absence of benzolamide. However, the increase in the horizontal cell membrane potential change did not lead to a greater shift of the Ca^2+^-current in cones, showing that the feedback efficiency has not increased, but may have even decreased slightly. This finding is inconsistent with the pH hypothesis, which predicts a larger shift in the cone Ca^2+^-current when carbonic anhydrase is inhibited with benzolamide.

### Intracellular acidification and ephaptic feedback

**Figure 10:**
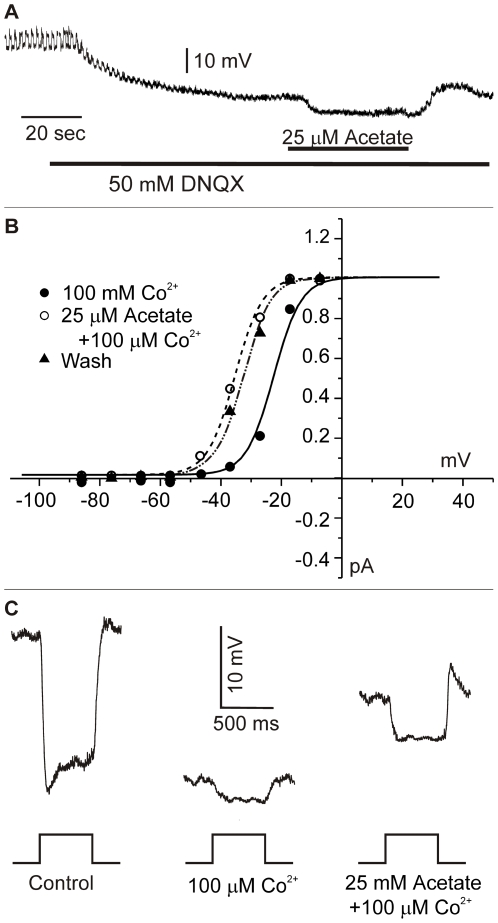
Effects of acetate on horizontal cells and cones in the isolated goldfish retina after chemical isolation. *A*, A horizontal cell was stimulated with full field 500 ms flashes of 500 nm light. Two time scales are given: one for the light flashes (top) and one for the whole trace (bottom). Removing synaptic input to a monophasic horizontal cell with 50 µM DNQX causes the cell to hyperpolarize. A subsequent application of acetate leads to a further hyperpolarization. *B*, Acetate exerts a direct effect on the cone Ca^2+^-current after pharmacologically isolating the cone from H-cell input with 100 µM Co^2+^. Boltzmann fits through activation function of the Ca^2+^-current in the presence of Co^2+^ (filled circles), after application of 25 mM acetate (open circles), and after washout (filled triangles). Boltzman fits were obtained as described in Fig. *3a*. In the presence of Co^2+^, acetate shifts the Ca^2+^-current to more negative potentials. *C*, Responses of a monophasic horizontal cell to 550 nm light (I = 0 log) in control solution (left), after application of 100 µM Co^2+^ (middle), and after subsequent application of 25 mM acetate (right). The low concentration of Co^2+^ hyperpolarizes the horizontal cell and strongly reduces the amplitude of its light response. The addition of 25 mM acetate depolarized the cell, and increased the amplitude of the light response, indicative of an acetate-induced increase in neurotransmitter release.

Our data suggest that application of HEPES, Tris and Acetate lead to intracellular acidification and to inhibition of feedback. Can these results be accounted for by the ephaptic hypothesis? It is important to recall that hemichannels (unpaired connexins) are a key component of the ephaptic feedback pathway. In this paper we have presented evidence that suggest that hemichannels are sensitive to low concentrations of HEPES. In addition, our experiments suggest that intracellular acidification leads to closure of hemichannels in horizontal cells [34] as well as in cell lines in which connexins are expressed heterologously [35], [36]. To determine whether intracellular acidification induced by acetate application had a similar effect on hemichannels in the isolated retina, we tested the effect of acetate when horizontal cells were chemically isolated from their cone input by the application of 50 µM DNQX. If acetate blocks hemichannels in the isolated retina, horizontal cells should hyperpolarize because the hemichannels have a reversal potential more positive than the horizontal cell resting membrane potential. We tested this in Fig. 10A. The retina was stimulated with 500 nm, 500 ms flash of full field illumination every 1 second. In response to 50 µM DNQX, horizontal cells hyperpolarized and lost their light responses. In this condition application of acetate hyperpolarizes horizontal cells even further. In the 6 cells tested in this way, the mean acetate-induced hyperpolarization was 5.8±1.0 mV (p<0.025). The simplest explanation for this result is that acetate leads to intracellular acidification and in that way to a pH-dependent closure of hemichannels in horizontal cells. It is important however to recognize that an inwardly rectifying potassium channel is active in the hyperpolarized operating range of the horizontal cells [37]. Most potassium channels reduce their conductance upon intracellular acidification [38]. However, if intracellular acidification caused the inward rectifying potassium channels to close, the horizontal cells would have depolarized, contrary to our experimental findings.

Tombaugh and Somjen [39] have shown that intracellular acidification affects L-type Ca^2+^-channels in hippocampus. This effect was accounted for by surface charge theory [40]. In this view, increasing the intracellular proton concentration neutralizes negative charges on the inside of the membrane leading to a shift of the Ca^2+^-current to negative potentials. In principle, Ca^2+^-channels in horizontal cells could also be affected by this sort of mechanism. However the effect of acetate on the Ca^2+^-current would have led to depolarization of horizontal cells, not to hyperpolarization.

Since we found that cones in our experiments also acidified, we studied the effect of acetate on the Ca^2+^-current of cones. Cones were pharmacologically isolated from horizontal cells by blocking the feedback pathway with 100 µM cobalt (Co^2+^). At low concentrations, Co^2+^ blocks feedback without directly affecting the feedforward signal from cones to horizontal cells [41]–[43]. Fig. 10B shows the Ca^2+^-current in the presence of 100 µM Co^2+^ (filled circles), after addition of acetate (open circles), and after washout (filled triangles). In the absence of a feedback signal, acetate shifted the Ca^2+^-current to more negative potentials (7.48±1.3 mV; n = 5; p<0.05), suggesting that, in addition to blocking hemichannels in horizontal cells, acetate exerts a direct effect on the Ca^2+^-current of cones most likely induced by the intracellular surface charge effect.

This result predicts that application of acetate in a condition when hemichannels are blocked will cause horizontal cells to depolarize, because of an acetate-induced increase in the Ca^2+^-current of cones and a resultant increase of neurotransmitter release. As suggested by Fig. 10C, blocking feedback from horizontal cells with 100 µM Co^2+^ led to a hyperpolarization of horizontal cells, and a loss of feedback induced roll-back responses. Subsequent application of acetate resulted in a depolarization of the horizontal cell and an increase in its light response (trace at right), but the roll-back did not re-appear. All 5 horizontal cells tested in this way behaved similarly. Although, the effects of application of Tris, HEPES and acetate can be accounted for by the ephaptic feedback hypothesis, we aimed at further testing this idea.

A crucial prediction of the ephaptic mechanism is that *any current source* within the synaptic cleft will contribute to the feedback response. This feature can be used to further distinguish between the pH and the ephaptic hypotheses. We had suggested previously that under conditions where part of the glutamate-gated channels on the horizontal cell dendrites are kept open by non-saturating concentrations of kainate, glutamate-gated channels can contribute to feedback from horizontal cells to cones [7], [9]. In these circumstances, the open channels of the glutamate receptors mimic the hemichannels. Thus, in conditions where hemichannels are blocked with acetate, feedback should become functional if a fraction of the glutamate channels are in the open state.

**Figure 11:**
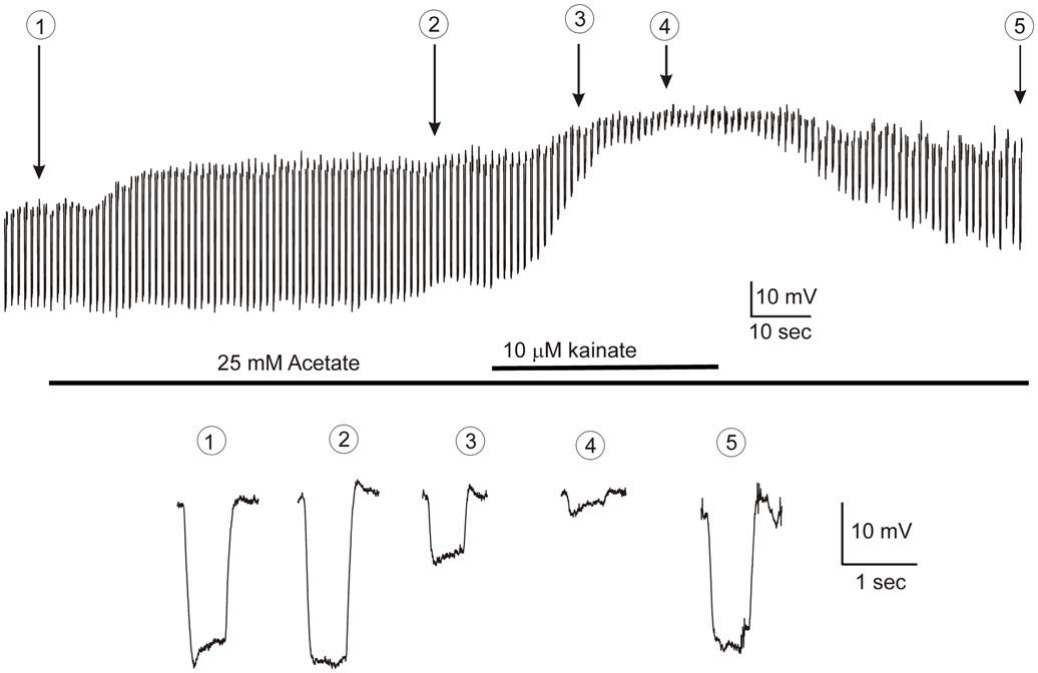
Glutamate-gated channels are able to mediate feedback in the presence of acetate in the isolated goldfish retina. Responses of a monophasic horizontal cell to 550 nm light (I = 1 log) during application of acetate and kainate. Expanded light responses at the time points indicated by the arrows, are given below. 1) light response in control with a feedback-induced roll-back response. 2) light-responses in the presence of acetate. The feedback-induced roll-back response has disappeared without significant changes in the light response amplitude. 3) light-responses after additional application of 10 µM kainate. Despite a reduction in the amplitude of the light response, the feedback-induced roll-back response reappears again after opening a fraction of the glutamate-gated channels with a non-saturating concentration of kainate. 4) Light responses after longer superfusion of kainate. As horizontal cells depolarize further, their light responses get smaller, but there is still a substantial feedback-induced roll-back response. 5) Light response after washout of kainate; note that the horizontal cell hyperpolarizes, the amplitude of the light response increases, and the roll-back response is again no longer present.

Fig. 11 shows light-induced responses of a monophasic horizontal cell to a 3000 µm spot in control conditions, in acetate, and in acetate with a non-saturating concentration of kainate (10 µM), followed by a washout phase. Note that the roll-back response of the horizontal cell light response (trace 1) is no longer evident in the presence of 25 mM acetate (trace 2). However, subsequent application of 10 µM kainate partially depolarized the cell, and despite the reduced response amplitude, the feedback-mediated roll-back reappeared (traces 3 and 4). The removal of kainate led to hyperpolarization of the horizontal cell, an increase of the light response amplitude, and a disappearance of the roll-back (trace 5). Similar results were obtained in all 8 cells tested. These results are consistent with an ephaptic feedback mechanism.

### Feasibility of ephaptic interactions between horizontal cells and cones

**Figure 12:**
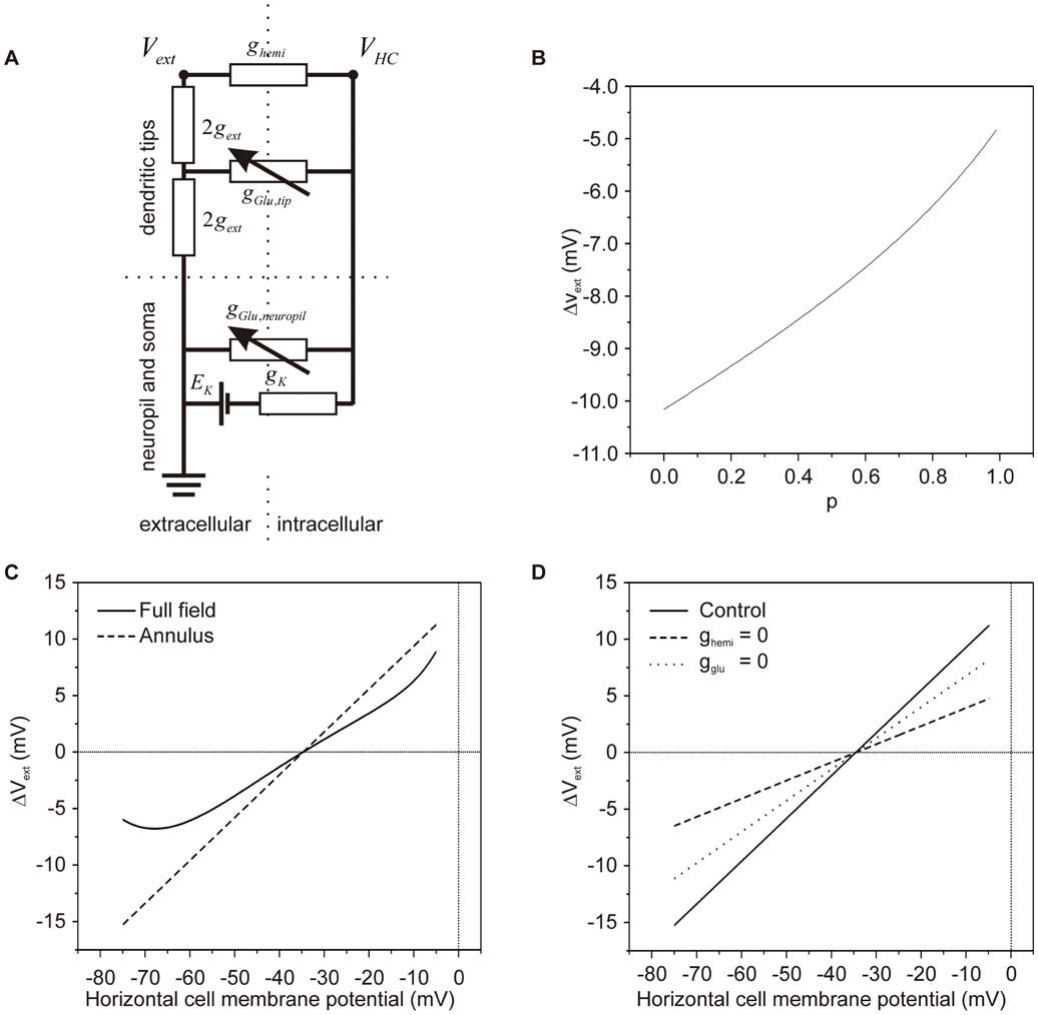
Model for ep[haptic feedback from horizontal cells to cones. *A*, Network of components essential to model ephaptic feedback from horizontal cells to cones. The ephaptic influence of the horizontal cell onto the cones is through changes in the extracellular potential of the synaptic cleft. 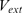 is the output of the model and affects the position of the Ca^2+^-current of the cone along the voltage axis and thereby modulates the amount of calcium flowing into the cone. The horizontal cell has two compartments, 1) the neuropil and soma, and 2) the dendritic tips. The neuropil and soma compartment has the following components: 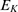, reversal potential of combined potassium and leak channels; 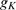, the combined potassium and leak conductance. 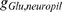, glutamate conductance in the neuropil and soma. The dendritic components are: 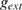, the conductance of the extracellular space in the synaptic cleft between cones and horizontal cells; 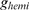, the hemichannel conductance, and 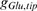, the conductance of the glutamate-gated channels present in the tip of the horizontal cell dendrites. *B*, The relation between the fraction of glutamate-receptors in the tip of the horizontal cell dendrite (*p*) and the size of the voltage drop in the synaptic cleft when horizontal cells are polarized from their resting membrane potential (−34.7 mV) to −71.4 mV. The smaller the fraction of glutamate-gated channels on the tips of the horizontal cells, the larger the horizontal cell polarization-induced voltage drop in the synaptic cleft. *C*, Voltage drop elicited in the synaptic cleft at the different membrane potentials of horizontal cells when stimulated with a full-field (solid line) or with an annulus (dashed line). When horizontal cells are maximally polarized in the full-field condition, a 6 mV potential drop is elicited; an annular stimulus gives a 15 mV potential drop. *D*, The relation between the horizontal cell membrane potential and the change in potential deep in the synaptic cleft for annular stimulation (solid line) and in a condition when all hemichannels are closed (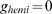) (dashed line) and when all glutamate gated channels are closed (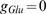) (dotted line). These simulations show that about 40% of the feedback response remains when hemichannels are closed, illustrating that glutamate gated channels contribute about 40% to the ephaptic feedback mechanism.

The feasibility of an ephaptic mechanism was challenged by Dmitriev and Mangel [44]. They used a simple resistive network to evaluate whether the physiology and morphology of the cone/horizontal cell synapse allows for physiologically relevant ephaptic interactions. We followed their model closely but we extended the model such that it becomes more in line with the physiology and morphology of horizontal cells (Fig. 12). The full model and the values of the relevant parameters are given in Text S1 and Tables S1 and S2. Four major differences with the model of Dmitriev and Mangel are: 1) The potassium channels in horizontal cells had a physiological potential dependence, 2) the *in situ* input resistance of horizontal cells was used instead of the input resistance of dissociated horizontal cells, 3) distributions of hemichannels, glutamate receptors and potassium channels based on immunohistochemical data were used and 4) physiological connectivity between horizontal cells and cones was implemented.

The model values for the various membrane conductances and reversal potentials were estimated following the procedure outlined in Text S1. The model has two free parameters: *p*, the fraction of glutamate gated channels on the tip of the horizontal cell dendrites, and *T*_,_ the tortuosity factor which describes the increase in diffusion constant due to the properties of the extracellular space (see Text S1). Such a tortuosity factor is about 1.5 [45], [46] but can be as high as 2.5 [47]. These values were estimated based on experimental data presented in the present paper.

The model was used to evaluate the relation between the horizontal cell membrane potential (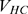) and the change in extracellular potential (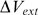) under a full field and annular stimulation. Full-field light stimulation leads to hyperpolarization of cones, a reduction of their glutamate release and a closure of the glutamate gated conductance on the horizontal cell dendrites (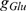). This leads to hyperpolarization of horizontal cells, an increase in the potassium conductance (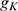), and an increase in current flowing through the hemichannels. This current will also flow through the extracellular space in the synaptic complex. This space has a finite conductance (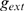), this current will induce a voltage drop which will result in a shift of the Ca^2+^-current in the cones to negative potentials. In this paper we have shown that full-field light stimulation hyperpolarizes horizontal cells 52.7 mV and shifts the Ca^2+^-current of cones 9.3 mV to negative potentials. These values could be reproduced by the model by slowing down diffusion in the synaptic cleft by 24% (free parameter *T* = 1.24) and assuming that 75% of the glutamate receptors were homogeneously distributed over the invaginating horizontal cell dendrites, and the remaining 25% of the glutamate receptors was present in the neuropil (free parameter *p* = 0.75) (Fig. 12B). These are realistic values. See the Text S1 for details.

Next we tested whether the model, with these values of *p* and *T*_,_ could reproduce a unique characteristic of feedback from horizontal cells to cones: i.e. in the range of physiological membrane potentials, 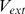 depends linearly on horizontal cell membrane potential (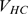) [48]. The solid line in Fig. 12C gives the relation between 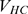 and 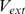 and shows that the model reproduces this linear relationship almost completely. Only at very hyperpolarized potentials does the curve deviate from linearity. This is most likely due to the strong activation of the potassium current in horizontal cells (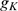) at these potentials.

Until now, only conditions where the retina was stimulated with full-field light were simulated. Often responses to annular stimulation are studied. In that case, the cone receiving feedback is not directly stimulated by light, but it receives feedback from horizontal cells that are hyperpolarized by the annulus. To simulate this condition,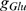 was kept constant and the relation between 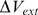 and 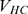 was determined (Fig. 12C). One would predict that feedback measured in the central cone is larger for annular stimulation than for full-field stimulation. For annular stimulation, current flowing through both 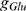 and the hemichannel conductance (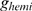) will increase with horizontal cell hyperpolarization, whereas for full-field stimulation the current through 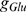 will reduce and the current through 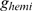
will increase. Fig. 12C confirms this prediction and shows that feedback becomes more pronounced for annular stimulation (dashed line) than for full field stimulation (solid line).

Both glutamate receptors and hemichannels contribute to the ephaptic interaction. Next the relative contribution in determining 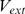 of these two conductances was determined. Since 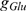 is diffusely-, and 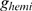
is focally- distributed (see Text S1), their effectiveness in generating ephaptic feedback will differ. 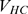 was varied in control conditions (Fig. 12D solid line), in conditions where 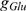 was set at zero (Fig. 12D, dotted line) and when 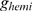 was set at zero (Fig. 12D dashed line). As can be seen from the figure, in conditions when hemichannels are not contributing, 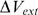 reduces to about 40% of the control value, indicating that for annular stimulation, the glutamate gated channels generate about 40% of the current essential for ephaptic feedback.

## Discussion

In this study, two very different views concerning the mechanism of negative feedback from horizontal cells to cones were evaluated: a pH-based mechanism and a hemichannel-mediated mechanism. The experimental data presented in this paper do not seem to lend support for the pH hypothesis, but are consistent with the presence of a hemichannel-mediated mechanism. In this regard, a key observation relates to the method typically used to test the pH hypothesis, namely, to observe the block of feedback after adding high concentrations of HEPES or Tris to the Ringer's solution. Although we confirmed that feedback was reduced by such manipulations, a more careful analysis revealed: 1) feedback cannot be blocked fully even with very high concentrations of HEPES or Tris, 2) that HEPES is the more effective inhibitor of feedback compared to Tris, although their buffering capacities are equal at the pH in which we worked (pH = 7.8), 3) that HEPES affects hemichannels directly, and 4) that artificial buffers lead to intracellular acidification.

Davenport et al. [14] suggested a correlation between the buffer capacity of the added buffer and the inhibition of feedback. However, the presence of carbonic anhydrase suggests that the pH buffer capacity of bicarbonate cannot be ignored. The pH buffer capacity of the various pH buffers in a solution must be added together to obtain the overall pH buffer capacity of a solution [49]. 0.4 mM HEPES, which induces half maximal inhibition of feedback, changes in our recording conditions the buffer capacity only 0.7%. These small changes in pH buffer capacity make it unlikely that such manipulations lead to significant change in a proton-mediated feedback.

On the other hand, we suggest that application of HEPES or Tris causes measurable intracellular acidification. A possible mechanism for such intracellular pH changes might be an increase in glycolysis and lactate production, both of which will decrease intracellular pH [50]. A large number of pH regulating systems exist in and around neurons and it is likely that pH is not homogeneous around cells. The pH hypothesis suggests that micro environments might exist where pH is regulated differently than elsewhere. The pH-regulating systems tend to keep the pH stable at a preferred value in these various domains. Preventing the existence of these micro-environments and cellular pH regulation through addition of pH buffers will likely activate the pH regulating systems further and may lead to increased metabolic activity, resulting in intracellular acidification. Such effects are not unprecedented. Several researchers have reported intracellular acidification after application of Tris or HEPES [50]–[52].

The results presented so far raise the question of whether intracellular acidification or extracellular pH buffering leads to the inhibition of feedback. The experiments with acetate were designed to discriminate between these two options. Application of 25 mM acetate leads to a strong decrease in intracellular pH but does not lead to an increase in extracellular buffer capacity. As a pH buffer, acetate is about 100 times less effective than HEPES in our recording conditions. The results of Fig. 6 show that acetate blocks feedback, suggesting that intracellular acidification is the mechanism that leads to a block of feedback.

How could intracellular acidification lead to block of feedback? Horizontal cells express connexin hemichannels on the tips of the dendrites that invaginate into the cone synaptic terminal. It has been proposed that these hemichannels are involved in feedback from horizontal cells to cones via an ephaptic interaction [7]. Intracellular acidification blocks hemichannels [34]–[36]. According to the hemichannel hypothesis, this should lead to an inhibition of feedback. This is fully in line with what was found.

Interestingly, HEPES is more efficient in inhibiting feedback than Tris, whereas they have about equal pH buffer capacity in our experimental conditions (pH 7.8; 4 mM HEPES: 2.0 mM versus 4 mM Tris: 2.1 mM). The reason for this difference in efficiency might be that application of HEPES affects hemichannels directly as well. At the very least, these experiments offer an alternative explanation for the key experiments favoring the pH hypothesis.

### Decreasing the endogenous pH buffer capacity

The present paper offers, however, a second argument independent of artificial pH buffer systems, indicating that protons are unlikely to mediate feedback. We used the pH-nose of the Ca^2+^-current in bipolar cells and cones to determine whether the inhibition of the endogenous buffer system by application of benzolamide was effective. The pH-nose on the Ca-current of both bipolar cells and cones becomes larger when one inhibits extracellular carbonic anhydrase. This suggests that the pH changes in the synaptic cleft in bipolar cells increase when the endogenous buffer becomes less effective. The pH hypothesis predicts that larger pH changes in the cone synaptic clefs should have increased the proton-mediated feedback. However, the opposite was found. Quantification of feedback with the kainite/DNQX protocol showed that feedback did not increase and may have even decreased slightly.

In principle a Na/HCO_3_ transporter could modulate the extracellular HCO_3_^−^ concentration in response to horizontal cell polarization and mediate in that way negative feedback from horizontal cells to cones. Blocking extracellular carbonic anhydrase with benzolamide would inhibit the production of extracellular HCO_3_^−^ and thus interfere with such a system and lead to a reduction of feedback. If this were the mechanism of negative feedback from horizontal cells to cones, then feedback should be strongly enhanced by intracellular acidification, because intracellular acidification leads to enhanced activity of the Na/HCO3 transporter [30]. The opposite was found.

### Blocking feedback and polarization of horizontal cells

We have shown that HEPES induced a hyperpolarization of horizontal cells and a decrease in the amplitude of light responses. Such hyperpolarization is expected to occur when one blocks feedback. The horizontal cell membrane potential is set by a number of parameters. The most important is the glutamatergic input from cones (feedforward pathway). This input depends on the membrane potential of the cones and the amount of feedback a cone receives (feedback pathway). This last parameter depends on the horizontal cell membrane potential making it a closed loop system. The resting potential of horizontal cells is approximately −30 mV by virtue of a balance between a feedforward pathway and a feedback pathway. Blocking feedback leads to a shift of the Ca-current to positive potentials and thus to a reduction of glutamate release which will induce a hyperpolarization of horizontal cells. Therefore, blocking feedback without any other change in the system should lead to a reduction of the glutamate gated conductance in horizontal cells and thus hyperpolarization [43].

Reports of the effect of HEPES on the membrane potential of horizontal cells are variable. Hirasawa and Kaneko [10] and Davenport et al [14] showed no change in horizontal cell membrane potential whereas Hare and Owen (1997) and Yamamoto at al [53] showed a strong depolarization with HEPES, and Hanitzsch and Küppers [54] showed strong hyperpolarization. How to account for these differences? In addition to the feedforward and the feedback pathway, the membrane potential of horizontal cells will be determined by other conductances as well. These conductances will, at least, include potassium channels and hemichannels. These channels are potentially affected by changes in intracellular pH. Potassium channels can reduce their conductance upon intracellular acidification [38] leading to an increase in depolarizing force on the membrane potential. Intracellular acidification leads also to the closure of hemichannels, which tend to depolarize horizontal cells. For the cone this will mean more glutamate release and for the horizontal cell this will mean a larger depolarizing drive. Since we are comparing the effect of HEPES on the horizontal cell membrane potential in various animal systems (primate, rabbit, salamander and goldfish), the differences in results might be accounted for by different relative contributions of the various systems to the membrane potential.

Finally, our experiments are performed in a condition where the GABAergic input to both horizontal cells and cones are blocked. Cones and horizontal cells in at least both fish and salamander have GABA_A_-receptors. Although GABA does not seem to be the major neurotransmitter that mediates the negative feedback signal to cones [55], changes in GABA will lead to changes in membrane conductance, membrane potential of cones and horizontal cells and in changes in receptive field size of horizontal cells [56]. We cannot exclude that depolarization due to the application of HEPES or Tris seen by Yamamoto et al. [53] is due to alterations in the GABAergic system.

HEPES and acetate both lead to intracellular acidification and to a block of feedback. However, acetate does not whereas HEPES does lead to hyperpolarization of horizontal cells. How can we account for this difference? First it is important to recall that HEPES and acetate lead to changes in intracellular pH via very different mechanisms. Secondly we have to realize that HEPES in addition to intracellular acidification inhibits hemichannels directly which leads to hyperpolarization of horizontal cells. This hyperpolarizing effect is not present in the acetate experiments.

### pH changes in the synaptic cleft of photoreceptors and bipolar cells

DeVries [31] and Palmer et al [19] showed that transient pH changes in the synaptic cleft lead to inhibition of the Ca^2+^-current of cones and bipolar cells. These pH changes are due to the low pH in the synaptic vesicles, estimated to be about 5.7 [57]. Fusion of vesicles with the membrane leads to a change in extracellular pH. This transient inhibition of the Ca^2+^-current can be blocked by application of 48 mM HEPES. Interestingly, 3 mM HEPES was ineffective in blocking these transients: i.e. the transients did not significantly differ from those measured in bicarbonate Ringer's solution [19]. This is in contrast to the effect of HEPES on feedback responses measured in cones, where 0.4 mM HEPES inhibits feedback half maximally and where 4 mM HEPES is a saturating concentration. This comparison suggests that the pH nose and negative feedback have a different dependence on HEPES and are thus not mediated via a common pathway, i.e., the extracellular pH.

In this paper we have shown that extracellular carbonic anhydrase is present in the outer and inner retina. Because of the presence of carbonic anhydrase, bicarbonate is a much more effective pH buffer than HEPES. So, why does bicarbonate not buffer the pH transients in the cone and bipolar cell synapses? A possible explanation could be that HEPES is a much faster buffer than bicarbonate even in the presence of carbonic anhydrase. This would mean that fast pH transients very close to the membrane, such as found by DeVries [31] and Palmer et al. [19], can occur in the synaptic cleft, but that sustained pH changes needed for a proton-mediated sustained feedback signal are fully buffered by bicarbonate. Our data support this hypothesis.

### The ephaptic feedback hypothesis

The present data support the notion that hemichannels are involved in negative feedback from horizontal cells to cones. We hypothesized that their involvement is via an ephaptic interaction. One key feature of an ephaptic mechanism is the fact that any current in the synaptic terminal should be able to mediate feedback responses. To test that, we have used kainate to cause sustained opening of a portion of glutamate receptors in horizontal cells in a condition where hemichannels were blocked by carbenoxolone, or by 100 µM Co^2+^ [7], [43]. In such conditions, feedback reappeared as soon as a portion of the glutamate receptors were opened. These experiments indicated that both the inhibition of feedback by carbenoxolone and by 100 µM Co^2+^ were not due to non-specific effects on the Ca^2+^-current of cones, but were an effect of blocking a current in horizontal cells. In this paper, similar results with intracellular acidification by acetate were presented, generating more support for an ephaptic mechanism.

The feasibility of an ephaptic mechanism has been questioned. Dmitriev and Mangel [44] concluded, based on a computational model, that the ephaptic interaction in the cone terminal is much too small to be of physiological relevance. We have scrutinized their assumptions and modified their model. Now it reproduces the essential features of the ephaptic feedback pathway and demonstrates that, under conditions of both full-field and annular illumination, the currents generated through the glutamate-gated channels and hemichannels of horizontal cells are sufficient to modulate the release of neurotransmitter from the terminals of cone photoreceptors. Moreover, for reasonable parameter values, this modulation can account for the measured negative feedback responses. In its present form, our model adds two essential features to the model developed by Dmitriev and Mangel [44], namely, the more widespread distribution of glutamate receptors and the non-linearity of the potassium channels on horizontal cells. Both have a major impact on the effectiveness of the feedback signals.

The non-linearity of the potassium current has several major implications for the ephaptic mechanism, which were recognized earlier by Byzov and Shura-Bura [8]. When horizontal cells start to hyperpolarize in response to the closure of their glutamate-gated channels, the potassium conductance will begin to activate. As a result, the horizontal cell will hyperpolarize to a greater extent than due solely to the closure of 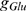; this leads, in turn, to larger light-induced responses, and a concomitant increase in the feedback response. Moreover, the potassium conductance, which might limit the total current flowing through the hemichannels in depolarized conditions, increases with hyperpolarization allowing more current to flow through the hemichannels, and thus provides a further enhancement of ephaptic feedback.

Dmitriev and Mangel [44] suggest that ephaptic feedback should be positive for full-field stimulation because the reduction of the current flowing through the glutamate receptors will exceed the increase of the current through the hemichannels. This would indeed be the case if these two current sources were located at the same location, i.e. at the tips of the horizontal cell dendrites. However, with the addition of the potential dependence of the potassium channels and the more diffuse localization of the glutamate receptors, which more accurately reflects their distribution *in situ*, the present model predicts that ephaptic feedback will always be negative.

### Conclusion

#### Multiple feedback pathways in the cone synapse

The feedback mechanism is obviously a complex process. The hypotheses we have discussed entail two novel forms of neuronal communication. A clear understanding of these mechanisms is still in its infancy, and methods to evaluate these types of communication have not evolved to the level available for more conventional neuronal interactions. The data presented in this paper lend support for the ephaptic nature of feedback, adding to the increasing body of evidence that physiologically significant ephaptic interaction can be present between neurons [58].

However, to strictly argue that neither GABA (a neurotransmitter system of importance in the outer retina) nor protons play a role in the communication in the outer retina is unwarranted at this time. Both appear to be involved, but do not seem to be mediating the fast inhibitory feedback system that underlies the center/surround organization of bipolar cells. GABA most likely either contributes weakly to negative feedback [55], or to act as a slow and global neuromodulator of negative feedback [59], [60]. Protons might be involved in fast, transient and very local modulation of the Ca^2+^-current of cones [31] and bipolar cells [19]. Finally, extracellular pH seems to change in a light/dark and circadian fashion [61], [62]. This opens the possibility that pH affects outer retinal processing also in a very slow and global way. Thus, it seems that there are a number of simultaneously active feedback pathways present in the cone synapse, all with different time constants and different spatial integration areas. Understanding how these pathways interact will be the challenge for the near future.

## Materials and Methods

### Experimental Animals

Goldfish, *Carassius auratus* (12–16 cm standard body length), were kept at 18°C under a 12-hour dark, 12-hour light cycle, and experiments were performed with fish that were between 6 and 9 hours into their light phase. All recordings from cones and horizontal cells were made in flat mounted isolated retinal preparations. Recordings from bipolar cell terminals were made in retinal slices. Properties of connexin hemichannels were studied in Xenopus Oocytes.

All experimental procedures adhered to the ARVO Statement for the Use of Animals in Ophthalmic and Vision Research, and conformed to the guidelines for the Care and Use of Laboratory Animals of The Netherlands Institute for Neuroscience acting in accordance with the European Communities Council Directive of 24 November 1986 (86/609/EEC).

### Experimental Procedures

#### Isolated retina preparation for cone and horizontal cell recordings

The fish were dark-adapted for at least 3 minutes, and all further steps in preparation were performed in the dark under deep red light illumination. After decapitation, an eye was enucleated and hemisected and most of the vitreous was removed with filter paper. The retina was isolated, placed receptor side up in a superfusion chamber (volume 0.75 ml) mounted on a Nikon Optiphot-X2 microscope (Nikon, Japan), and superfused continuously (1.5 ml/min) with a Ringer's solution of which the pH was continuously measured. The compositions of the different Ringer's solutions used are given in Table 1. All chemicals were obtained from Sigma-Aldrich (St Louis, MO), except for SKF89976A (a kind gift from Smith Kline Beecham Pharmaceuticals) HEPES and Tris (Merck, Darmstadt, Germany) and benzolamide (a kind gift from Dr E. Swenson). The solution was continuously gassed with a mixture of 2.5% CO_2_/97.5% O_2_. Minor adjustments to the amount of CO_2_ were made such that the pH was exactly 7.8. 4-(2-hydroxyethyl)-1-piperazineethanesulfonic acid (HEPES) or tris(hydroxymethyl)aminomethane (Tris) was added to a solution with 20% less NaCl. After equilibrating the solutions with the same CO_2_/O_2_ gas mixture that was used to gas the control Ringer's solution, the pH was adjusted with NaOH or HCl, and the osmolarity was adjusted to the same value as the control Ringer's solution (248 mOsmol) by adding NaCl. This procedure ensured that the HEPES and Tris-containing Ringer's had equal osmolarity and pH as the control Ringer's solution. During all electrophysiological experiments with the isolated retina, GABAergic transmission was blocked by either 200 µM picrotoxin (PTX) or 50 µM SKF89976-A (Smithkline & Beecham French Laboratory).

**Table 1:** Ionic composition of solutions used in this study.

	NaCl	KCl	CaCl_2_	MgCl_2_	HEPES	Tris	NaHCO_3_	glucose	NaAcetate
Control	102	2.6	1	1	–	–	28	5	–
0.4 mM HEPES	112.8	2.6	1	1	0.4	–	16.8	5	–
4 mM HEPES	109.2	2.6	1	1	4	–	16.8	5	–
20 mM HEPES	93.2	2.6	1	1	20	–	16.8	5	–
48 mM HEPES	65.2	2.6	1	1	48	–	16.8	5	–
4 mM Tris	109.2	2.6	1	1	–	4	16.8	5	–
20 mM Tris	93.2	2.6	1	1	–	20	16.8	5	–
Acetate	77	2.6	1	1	–		28	5	25

#### Retinal Slice Preparation for bipolar cell recordings

Retinal slices (250 µm) were prepared from goldfish (*Carassius auratus*; 8–16 cm) as described previously [19]. Slices were transferred to the recording chamber (2 ml) and continuously perfused with oxygenated (95% O_2_, 5% CO_2_) Ringer's solution (2–3 ml/min). The control solution contained the following (in mM): 100 NaCl, 2.5 KCl, 1.0 MgCl_2_, 25 NaHCO_3_, 0.2 ascorbate, 2.5 CaCl_2_, 12 glucose, pH 7.45 (NaOH), mOsM 260. Drugs were bath applied in the perfusion solution. Recordings from the slice preparation were done at room temperature (21–23°C) under room light following 1–2 hr dark adaptation. Slices were viewed with infrared differential interference contrast imaging through a 40× water-immersion objective coupled with 2× premagnification (Optovart; Zeiss, Oberkochen, Germany) and a CCD camera (C79; Hammatsu, Tokyo, Japan). Bipolar cell terminals were identified by their position in the slice, shape, and size (6–10 µm diameter). Capacitance responses and depolarization evoked Ca^2+^-currents were used to confirm bipolar cell identity once whole-cell recordings had been established. Isolated bipolar cell terminals, with axons cut during the slicing procedure, were identified by their single-exponential capacitive current response to a 10 mV hyperpolarization from −60 mV [19]. Isolated terminals were also identified by their small membrane capacitance (3–7 pF), small leak current (<50 pA), and large input resistance (1–3 GΩ). We only used isolated bipolar cell terminals with severed axons in this study. Membrane capacitance measurements were performed with the EPC-9 patch clamp amplifier as described in [19].

#### Drug Application

Drugs were bath applied in Ringer's solution. Benzolamide was dissolved in water with drop-wise addition of NaOH and stored in stock solution of 100 mM at −20°C. Stock solution was diluted 1∶1000 into Ringer's solution containing 100 µM picrotoxin to block GABA receptors. The pH was adjusted to 7.45 with HCl and the final osmolarity was adjusted to 260 mOsm. Benzolamide was allowed to perfuse into the slice for at least 1–10 min prior to recording. Each bipolar cell terminal recording was done either by washing-in or washing-out benzolamide in the presence of 100 µM picrotoxin.

### Voltage clamp measurements of cone responses

#### Optical stimulator

A 450 W Xenon-lamp supplied two beams of light that were directed to the preparation after passing through Uniblitz VS14 shutters (Vincent associates, USA), neutral density filters (NG Schott, Germany), and a series of lenses and apertures. Feedback-induced responses to 500 ms, 3000 µm spot stimulation were measured in cones at different potentials while the cone light conductance was continuously saturated with a 20 µm spot. The 20 µm spots were projected through the 40× water immersion objective (N.A. = 0.55) of the microscope, and the 3000 µm spots were projected through the microscope condenser (N.A. = 1.25). For experiments with cones, only white light stimuli were used; light intensities are expressed in log units of attenuation relative to the maximum luminance of 4_*_10^3^ cd/m^2^.

#### Electrodes and recording equipment

Pipettes were pulled from borosilicate glass (GC150TF-10 Clark, U.K.) with a Sutter P-87 micropipette puller (Sutter Instruments Company, U.S.A.); the impedances ranged from 3 to 6 MΩ when filled with pipette medium and measured in Ringer's solution. The standard patch pipette medium contained [in mM]: KCl [10], D-Gluconic-K [96], MgCl_2_ [1.0], CaCl_2_ [0.1], EGTA [5.0], HEPES [5.0], ATP-K [5.0], GTP-Na_3_ [1.0], 3′: 5′-cGMP-Na [0.2], phosphocreatine-Na_2_ [20], creatine phosphokinase [50 units/ml]. In experiments with the standard patch pipette medium, E_Cl_ was calculated to be −55 mV. The pH of the pipette medium was adjusted to 7.25 with KOH. The electrodes were mounted on a MP-85 Huxley/Wall-type micromanipulator (Sutter Instruments Company, U.S.A.) and connected to a Dagan 3900A Integrating Patch Clamp (Dagan Corporation, U.S.A.). The liquid junction potential was measured with a patch pipette filled with the pipette medium, and positioned in a bath filled with pipette medium. The reference electrode was filled with 3 M KCl. After the potential was adjusted to zero, the bath solution was replaced with Ringer's solution. The resulting potential change was considered the junction potential, and all data were corrected accordingly. For E_Cl_ −55 mV, the junction potential was 8.5 mV. The preparation was illuminated with infrared light (λ>850 nm; Kodak Wratten filter 87c, USA), magnified with a Nikon 40× water immersion objective (N.A. = 0.55) modified for Hoffman modulation contrast, and viewed on a video camera (Philips, The Netherlands). Data acquisition, and control of the patch clamp and optical stimulator were done with a CED 1401 AD/DA converter (Cambridge Electronic Design Limited, U.K.) and a MS-DOS based computer system.

### Intracellular measurements of horizontal cell responses

#### Optical stimulator

The optical stimulator consisted of 2 beams from a 450 W Xenon light source, and a pair of circular neutral density filters (Barr & Strout, UK). The full-field chromatic light stimuli were projected onto the retina through a 2× objective lens (N.A. = 0.08) of the microscope. To classify the horizontal cell spectrally, a monochromator (Ebert, USA), and interference filters with a bandwidth of 8±3 nm (Ealing Electro-Optics Inc., U.S.A.) were used. The light intensities are expressed in log units relative to 4_*_10^16^ quanta sec^−1^ m^−2^. The intensities of the 550 nm and the 650 nm were respectively 0.4 and 0.2 log units lower than the intensity of the 600 nm stimuli.

#### Electrodes and recording equipment

Microelectrodes were pulled on a horizontal puller (Sutter P-80-PC; San Rafael, USA) using aluminosilicate glass (OD = 1.0 mm, ID = 0.5 mm; Clark, UK), and had impedances ranging from 300–400 MΩ when filled with 3 M KCl. The intracellular recordings were made with a WPI S7000A microelectrode amplifier system (World Precision Instruments, USA), recorded on paper (Graphtec Linearcorder, Japan), and sampled using an AD/DA converter (CED 1401, Cambridge Electronic Design, UK) coupled to a Windows based computer system.

### Measurements of proton inhibition of Ca^2+^-current

#### Electrophysiology

Whole-cell voltage-clamp recordings of bipolar cells were performed with 5–10 MΩ patch pipettes pulled from 1.5 mm OD thick-walled borosilicate glass (World Precision Instruments, Sarasota, Florida) with a Sutter (Novato, California; model P-97) puller. Pipettes were coated with dental wax (Cavex, West Chester, PA) to reduce pipette capacitance and electrical noise, and filled with solution containing the following (in mM): 60 Cs-gluconate, 40 CsCl, 28 HEPES, 10 TEACl, 3 Mg-ATP, 1.0 Na-GTP, and 2.0 EGTA, adjusted to pH 7.2 with CsOH. The osmolarity was adjusted to 250 mOsm. Cells with series resistance >30 MΩ were excluded from any further evaluation. Data acquisition was controlled by Pulse software (HEKA Elektronik, Lambrecht/Pfalz, Germany) and signals were recorded with a double EPC-9 (HEKA Elektronik) patch-clamp amplifier. The sampling rate and low-pass filter setting were 10 and 2.8 kHz, respectively. Capacitance measurements were performed by the “sine+DC” method, in which a 1 kHz sinusoidal voltage command (30 mV peak-to-peak) was added to the −60 mV holding potential for the first and last 100 ms of each trace. The resulting current was analyzed at two orthogonal phase angles by the EPC-9 lock-in amplifier. Ca^2+^-current and exocytosis were evoked by a 100 ms step to −30, −20, or −10 mV.

#### Analysis

The Ca^2+^-current in bipolar cell terminals is transiently inhibited by protons co-released with glutamate, resulting in a so-called “pH-nose” (Palmer et al., 2003). The total charge transfer of the “pH-nose” was calculated by scaling the Ca^2+^-current to match the peak amplitude of a current obtained following complete rundown of exocytosis. The pure Ca^2+^-current was then subtracted from the trace being analyzed, and the area of the resulting trace for the first 20 ms following depolarization was calculated. These steps were performed using a custom “IgorPro” software procedure. Capacitance jumps (ΔC_m_) were calculated by subtracting the mean pre-depolarization capacitance from the mean post-depolarization capacitance. Linear regression and statistical analysis were performed with “Prism4” software.

### pH imaging using two-photon microscopy

An upright Nikon Eclipse 600FN equipped with a D-Elcipse C1 confocal scanhead was used for the two-photon imaging experiments. A Mai Tai laser (Spectra-Physics, USA) was directly coupled to the scan head. Laser intensity was controlled by a 350-80 LA, BK Pockel cell (ConOptics, CT). The laser was tuned to 830 nm yielding an effective excitation wavelength of about 415 nm. A non-descanned detector was mounted on the fluorescence port of the microscope. The output of the photomultiplier tube (PMT) (R105UH, Hamamatsu, Japan) was fed into the acquisition board of the Nikon D-Eclipse C1. The photomultiplier was not sensitive to light above 625 nm. All light below 650 nm was directed to the PMT. When BECEF is excited blow 439 nm an increase in pH yield an increase in fluorescence light (<600 nm). BCECF-AM was only used to image pH changes and no attempt was made to convert fluorescence to absolute pH. BCECF-AM was dissolved in 250 µl DMSO containing 20% Pluronic acid to enhance dye loading. This solution was added to 2.5 ml Ringer's solution. In this loading solution, the final concentration of BCECF-AM was 32.2 µM, and of Pluronic acid 2%. An isolated retina was incubated for at least 45 min in the loading solution and washed for 45 min with control Ringer's solution to allow the cleavage of the AM group from the dye. The isolated retina was mounted with the receptor layer down in a superfusion chamber (volume 0.5 ml) and continuously superfused (1.5 ml/min) with a Ringer's solution (pH 7.8) gassed with 97.5% O_2_ and 2.5% CO_2_. pH of the Ringer's solution was measured continuously. A 60× water immersion objective (N.A. 1.00; Nikon, Japan) was used for all the imaging. Stacks of 10 images (step in Z-direction was 1 µm) centered around the synaptic terminals of the cones or around the horizontal cell layer were collected every 1 or 2 min. Stacks were collapsed and fluorescence in cone synaptic terminal or in horizontal cell soma was determined by identifying regions of interest (Fig. 4A). Since both the cone terminals and horizontal cell soma occupy most of the space in their respective layers, and by selecting the regions of interest prevented major contribution of fluorescence of other cell types such as Müller cells. Data were analyzed offline using Image-Pro 6.2 (Media Cybernetics Inc, MD, USA).

### Xenopus Oocyte recording and connexin expression

Xenopus oocytes were either obtained from Ecocyte (Castrop-rauxel, Germany) or from our own facility. The oocytes obtained from Ecocyte were handled according to the companies specifications. The procedure used for the oocytes obtained for our own facility have been described previously [35]. Briefly, oocytes were removed from gravid *Xenopus laevis* females (Xenopus One, Dexter, MI), and defolliculated by incubation in a calcium-free modified Barth's (MB) solution containing collagenase (2.5 mg/ml) for 2 hours under constant agitation. Stage V-VI oocytes were selected, and stored at 15°C in MB containing [in mM]: NaCl [88], KCl [1], NaHCO_3_ [2.4], HEPES [15], Ca(NO_3_)_2_ [0.33], CaCl_2_ [0.40], glucose [5], and MgSO_4_ [0.82]; 10 mg/L gentamycin (Gibco/BRL) was added, and the solution titrated with NaOH to pH 7.8. A plasmid containing the coding sequence of zebrafish Cx55.5 [22] was subcloned into the BamH1 site of the pCS2+ expression vector (generously provided by Dr. Thomas White, SUNY, Stony Brook, NY). The construct was linearized with the Not1 restriction endonuclease, and capped mRNA was transcribed in vitro with SP6 RNA polymerase using the mMessage mMachine (Ambion inc., Austin, TX) according to the manufacturer's instructions.

The effects of various HEPES concentrations on hemichannel currents were tested 48–72 hrs after injecting each oocyte with 46 nl of an aqueous solution containing a mixture of 50 ng Cx55.5 cRNA and 10 ng of an antisense oligomer to nucleotides 128–151 of the coding region of Cx38, the endogenous connexin of *Xenopus* oocytes. For the injections a nanoject II (Drumond, Broomall, PH, USA) was used. Cells that were injected with the antisense oligonucleotide alone served as controls. The oocytes were stabilized on a 0.5 mm nylon mesh in a Lucite chamber (volume = 0.7 ml), and superfused with test solutions through a multiport gravity feed system (MP6 manifold, Warner Instrument Corp., Hamden, CT); complete fluid exchange occurred in <8 sec.

Hemichannel currents were recorded using a two-electrode voltage clamp [63]. Current and voltage electrodes were pulled to resistances between 0.7 and 1.5 MΩ when filled with 3 M KCl, and connected to the input stages of either a GeneClamp 500B amplifier (Axon Instruments, Foster City, CA) or an Oocyte clamp (OC-725C, Warner inst, Hamden, CT, USA). Experimental protocols were controlled by pClamp 8 software through a Digidata 1322A acquisition interface (Axon) or by Signal 3 and a CED micro1401 acquisition interface (CED ltd. Cambridge, UK), Details about stimulus protocols are given in the text. I-V curves were plotted in Clampfit 8 (Axon) or Signal 3 (CED) and the numerical values representing each trace were entered in Microsoft Excel or Origin 7.5 (Microcal Inc. Northhampton, MA). Current amplitudes at 10 mV intervals were averaged, and transferred for graphical presentation to software programs in Origin 6 or Origin 7.5 (Microcal Inc. Northhampton, MA).

### Immunohistochemistry

#### Gel electrophoresis and immunoblot

Goldfish retinas were homogenized with a Teflon pestle in ice cold phosphate buffered saline (PBS), pH 7.4, containing a tablet of protease inhibitor cocktail (Boehringer Mannheim GmbH, Germany), one tablet per 50 ml PBS. After homogenization, a Tris-buffered sample (pH 6.8) solution was added with final concentration: SDS 2%, β-Mercaptoethanol 5%, Glycerol 10%, Bromphenolblue 0.005%. The samples were boiled for two minutes, centrifuged for 10 min at 14.600G, and supernatants were stored at −70° C until use. The samples were boiled for 2 minutes (3x), cooled, and then spun in a microfuge at 14.600G. Samples were subjected to sodium dodecyl sulphate polyacrylamide gel electrophoresis. Samples were run through a polyacrylamide stacking gel at 20 mA and through a 13% polyacrylamide gel running at 30 mA. Protein standards (Bio-rad Laboraties, BV, Veenendaal, The Netherlands) were run in adjacent lanes. Gels were electroblotted on PolyVinylideneDiFluoride blot membrane (Millipore, Amsterdam, The Netherlands) overnight at a constant current of 80 mA. The membrane was rinsed in a Tris buffer (0.5 M) containing NaCl (1.5 M) and 5% Tween 200. Afterwards, the membrane was blocked in the same buffer containing 2% dry milk for 1 hour, then incubated in the primary antibodies against carbonic anhydrase XIV (1∶100–500) for 1 hour, and washed in the Tris buffer. The membrane was then incubated in an HRP-conjugated goat anti rabbit IgG (Santa Cruz Biotechnology Inc, Santa Cruz, CA). After washing in PBS, the immunoreaction was visualized on the membrane paper by enhanced chemiluminescence (ECL, Amersham, Arlington Heights. IL) using Kodak film; exposure times were 1–5 minutes.

#### Light microscopy

Light adapted goldfish were transected cervically in dim room light. The eye was isolated, the cornea and lens were removed and the eyecup was cut in half along the dorsal-ventral axis. Half-eyecups were placed vitreous side down on a Millipore filter (Millipore BV, Amsterdam, the Netherlands) that was placed on a filter holder. Suction was used to remove the vitreous; the sclera and retinal pigment epithelium were peeled away. The retinas were fixed in 0.1 M phosphate-buffered 4% paraformaldehyde (pH 6.5) for 15 min, and subsequently fixed in 0.1 M sodium carbonate buffered 4% paraformaldehyde (pH 10.4). After rinsing in a 0.1 M phosphate buffer pH 7.4, cyroprotection was performed in PB containing: 12.5% sucrose for 30 min and 25% sucrose for 1–2 hours. All of these procedures were performed at room temperature. The pieces of retina still attached to the filter were embedded in Tissue Tek in an aluminum foil boat and frozen in dry ice. Thick sections (8–10 µm) were mounted on vectabond coated slides, dried and stored in a freezer at −20°C. The retinal sections were washed in phosphate buffered saline (PBS) pH 7.4, 10 min 2X, and blocked in 2% normal goat serum (NGS) in PBS for 20 min. Sections were incubated overnight with primary antibodies against carbonic anhydrase XIV (1∶500), the glutamate receptor subunit GluR2 (Chemicon International, Temecula, CA) (1∶100), or PKCα (Sigma-Aldrich, St Louis, MO) (1∶500) at 4°C in PBS containing 0.3% Triton X-100 and 5% NGS. To visualize the primary antibodies, sections were incubated, after washing in PBS, in Goat anti Rabbit Cy3 (Jackson Lab, Weat Grove,PA) and Goat anti Mouse Alexa-488 (Invitrogen Molecular Probes, Carlsbad, CA), rinsed again in PBS, coverslipped in Vectashield and stored at 4°C. Sections were observed on a Leica DMRD fluorescence microscope equipped with a filter set for Cy3. Sections for double label experiments were examined on an inverted Zeiss Axiovert 100 M microscope equipped with the LSM Meta 510 Laser Scanning Confocal module. Pre-adsorption was accomplished by mixing the antigen with the primary antibody at a 20 fold molar excess overnight at 4°C. Incubation proceeded as described above using the same concentration as used for the primary antibody. The primary antibody against carbonic anhydrase XIV was kindly provided by Dr Waheed [64], the antigen for the pre-adsorption test was kindly provided by Dr Sly. Light micrographs were acquired as TIFF files directly from the Leica and Zeiss Microscopes. All TIFF files were optimized for brightness and contrast in Adobe photoshop 7.0.

### Calculating pH buffer capacities

The buffer capacity of a solution with a pH above 2 and below 11.5 can be calculated using equation (1).
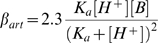
(1)

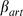
is the buffer capacity of the buffer, 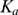 is the dissociation constant of the buffer, and [*B*]  = the buffer concentration. This equation only holds for “closed” buffer systems such as HEPES and Tris. In such systems the total amount of buffer is fixed. In a bicarbonate buffer system (Equation 2) the amount of buffer is not fixed since 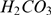 can be generated form 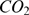 and 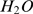, and is called an “open” buffer system. 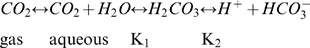
(2)The buffer capacity of the bicarbonate buffer system is given by equation (3) [30]. 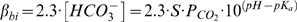
(3)

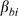
 is the buffer capacity of the bicarbonate buffer, 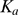 is the dissociation constant of the reaction between 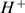
and 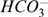 (6.35), *S* = the solubility of 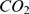 in water (38.7 mM/bar at 20°C and 25.5 mM/bar at 37°C), and 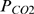 is the partial pressure of 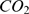. The fact that the amount of buffer is not fixed makes this buffer system a very good buffer at physiological pH. One interesting consequence is that with increasing alkalinization, more 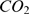 will dissolve resulting in a higher 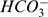 concentration leading to an increase in buffer capacity. This particular feature makes bicarbonate a very good pH buffer at physiological pH values [30], [49]. Note that this buffer only works quickly if carbonic anhydrase, the enzyme that catalizes the hydration of 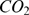, is present and that the buffer capacity of the bicarbonate system depends only on pH and on 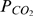. The buffer capacity of a solution containing more than one pH buffer is given by the sum of the buffer capacities of the various pH buffer systems in the solution. For simplicity, we only consider bicarbonate, HEPES and Tris and ignore other buffer systems such as phosphate buffers because these will only contribute modestly. The total buffer capacity of the Ringer's solution 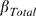 is then given by equation (4).
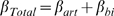
(4)The 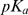 values used for the various buffers are given in Table 2 and the buffer capacities of the various solutions used is given in Table 3.

**Table 2:** Dissociation constants of pH buffers.

Buffer	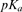
HEPES	7.48
Tris	8.06
Bicarbonate	6.35
Acetate	4.76

**Table 3:** Buffer capacities of Ringer's solutions.

	pH 7.8; t = 20°C; 2.5% CO_2_		
	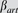 (mM)	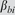 (mM)	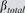 (mM)
Control	0	62.7	62.7 (100.0%)
0.4 mM HEPES	0.2	62.7	62.9 (100.3%)
4 mM HEPES	2.0	62.7	64.7 (103.2%)
20 mM HEPES	10.1	62.7	72.8 (116.1%)
48 mM HEPES	24.2	62.7	86.9 (138.5%)
4 mM Tris	2.1	62.7	64.8 (103.4%)
20 mM Tris	10.5	62.7	73.3 (116.8%)
25 mM Acetate	0.1	62.7	62.8 (100.1%)

### Measures of feedback

Feedback can be studied at various locations in the outer retina. In the present work, the effects of feedback in both cones and horizontal cells were measured. Feedback from horizontal cells to cones shifts the Ca^2+^-current of the cone to more negative potentials [4], [10], [13]. In a voltage-clamped cone, this shift can be seen as an increase in the Ca^2+^-current, which leads to an increase in glutamate released by the cone. The light induced shift of the Ca^2+^-current will be considered as a measure of feedback. The shift of the Ca^2+^-current was obtained by leak subtracting the cone I-V relationship; the leakage current was estimated from the linear portion of the I-V curve, between -109 and -89 mV. Half maximal potentials (a measure of activation) were derived from the I-V relation from −109 mV to the peak of the current response. To quantify feedback in cones, the maximal inducible shift of the Ca^2+^-current was determined by depolarizing horizontal cells with 30 µM kainate, and hyperpolarizing them with 50 µM DNQX. These measures of feedback will be considered as direct measures of feedback.

The monophasic horizontal cell hyperpolarizes in response to all stimulus wavelengths, and the resultant feedback-induced increase in glutamate release can be seen as a secondary depolarization, or roll-back from the response peak [65]–[68]. The size of the roll-back was determined as the difference between the peak of the response and its amplitude at 500 ms after the onset of the stimulus. This will be considered as an indirect measure of feedback. Biphasic horizontal cells show a depolarizing response to deep red light stimulation due to feedback [5], [66], [68]–[70]. This will be considered as a direct measure of feedback.

For quantitative purposes, the roll-back in the response of monophasic horizontal cells, the amplitude of the depolarizing response of the biphasic horizontal cell, and the maximal feedback-induced shift of the Ca^2+^-current of cones were taken as measures of feedback.

### Statistics

Data are presented as means±standard error of the mean. Significance was determined using the Student's-t test or an UNANOVA. p≤0.05 was considered as significant.

## Supporting Information

Text S1 (0.27 MB DOC)

Table S1 (0.05 MB DOC)

Table S2 (0.04 MB DOC)
