# Supplementary material for: Hemichannel-Mediated and pH-Based Feedback from Horizontal Cells to Cones in the Vertebrate Retina
Source: PLoS One. 2009 Jun 30;4(6):e6090. doi: 10.1371/journal.pone.0006090 (PMC2699542; doi:10.1371/journal.pone.0006090)
Supplement: Text S1 — (0.27 MB DOC) [file pone.0006090.s001.doc]

## Text S1

## A theoretical analysis of the ephaptic feedback mechanism

### Model description

A simple conductive network was used to evaluate whether the physiology and morphology of the cone/horizontal cell synapse allows for physiologically relevant ephaptic interaction. The interaction between one cone and one horizontal cell is considered, and a scaling procedure is used to obtain the behavior of the cone/horizontal cell network. The horizontal cell is modeled as three conductances with their associated reversal potentials; the hemichannel conductance ($g_{hemi}$), the glutamate gated conductance ($g_{Glu}$) and a potassium conductance ($g_{K}$). $g_{hemi}$and $g_{Glu}$are located on the dendrites and $g_{K}$at the soma of the horizontal cells; the reversal potentials for $g_{hemi}$and$g_{Glu}$are 0 mV and that for$g_{K}$is$E_{K}$. In the dark, the horizontal cell membrane potential ($V_{HC}$) is more positive than $E_{K}$, and current will flow from$g_{K}$into$g_{hemi}$and$g_{Glu}$via an extracellular resistive pathway in the synaptic complex whose conductance is ($g_{ext}$). This current will generate a voltage drop over $g_{ext}$, making the potential deep in the synaptic cleft ($V_{ext}$) slightly negative. The light-induced closure of$g_{Glu}$causes the horizontal cell to hyperpolarize, resulting in a change in the current through $g_{ext}$, and a greater negativity in$V_{ext}$. The fall in $V_{ext}$ serves to depolarize the cone membrane locally, modulate the cone Ca^2+^-current, and increase the release of neurotransmitter.

### Localization and properties of the conductances

$g_{ext}$: The current through the intersynaptic space encounters a resistance $R_{ext}=\frac{1}{g_{ext}}$. Based on a morphological reconstruction [1,2] we have determined that $R_{ext}$ can be as large as 60 MΩ. This value was also used by Dmitriev and Mangel [3]. However, physical constraints that limit diffusion have not been taken into account in these estimates. To deal with that, we introduce an additional tortuosity factor $T$and used $R_{ext}=T*60M\Omega$ [4–6].

$g_{hemi}$: The conductance through hemichannels, located focally at the tips of the horizontal cell dendrites [2,7,8], has a linear I-V relation with a reversal potential near 0 mV [8].

$g_{Glu}$: Glutamate-gated conductances are distributed diffusely along the horizontal cell dendrites of the synaptic terminal ($g_{Glu,tip}$) as well as in the neuropil directly below the cone terminals ($g_{Glu,neuropil}$) [9]. The relation between$g_{Glu,tip}$, $g_{Glu,neuropil}$and $g_{Glu}$is given by equation (1) where variable$p$represents the fraction of $g_{Glu}$ located on the horizontal cell dendrites.

$\begin{matrix} g_{Glu}=g_{Glu,tip}+g_{Glu,neuropil} \\ g_{Glu,tip}=p\cdot g_{Glu} \\ g_{Glu,neuropil}=(1-p)\cdot g_{Glu} \end{matrix}$ (1)

To model the diffuse nature of$g_{Glu,tip}$, this conductance is positioned half way between the base of the cone pedicle and the tip of the horizontal cell dendrite. In Fig 12A this is represented by the two $2\cdot g_{ext}$conductances.

$g_{K}$: The potassium conductance is distributed uniformly over the entire horizontal cell surface [10]. The voltage dependence of the combined steady state potassium conductance and the leak conductance were derived from the I-V relation of dissociated horizontal cells [11]. These two conductances will be combined and referred to as $g_{K}$. The constants 3.5 and ½ are additional scaling factors that will be introduced in the next section.

(2)

To simplify the analysis, no delayed rectifying potassium currents or Ca^2+^-currents were included; these currents will not contribute significantly to the total current of the horizontal cells in their normal operating range. The electrical coupling between horizontal cells is not included, and the internal resistance of the horizontal cell dendrites is neglected.

### Estimation of g_K_

To generate quantitative values the model needs an estimate$g_{K}$*in vivo*. This value is not directly available, but can be estimated based on measurements on dissociated horizontal cells. To convert the magnitude of the potassium current as measured in dissociated horizontal cells to that in horizontal cells *in situ* requires estimates of the surface areas of the cells under these two conditions. However, the area of the horizontal cell soma, the number of cones contacted by each horizontal cell, and the number and length of the dendrites vary amongst the three classes of horizontal cells in goldfish. Based on the data of Stell and Lightfoot [12] these values and the calculated surface area of the various horizontal cell types (soma plus dendrites) *in situ* are given in Table S1.

Now according to the findings of Yagi and Kaneko [13], the potassium current density of the axon is comparable to that of the soma, whereas the potassium current on the axon terminal is 1/20 that of the soma. By correcting the surface area for these current densities one can obtain an estimate of the effective surface area for the distribution of the potassium and leak conductance on a horizontal cell complete with dendrites, axon and axon terminal. By assuming that horizontal cells lose their axons, axon terminals and 20 % of their dendrites one can obtain the relative size of the potassium current in dissociated horizontal cells. Table S1 shows that the effective density of potassium channels *in situ* is 3.1 to 3.9 times greater than in dissociated horizontal cells; we used a factor of 3.5 which appears in equation 2 as a scaling factor.

### Iso-potentiality

Dmitriev and Mangel [3] question whether the currents through individual HC dendrites invaginating one cone may be summed or not. If not then the voltage change induced by an ephaptic mechanism should be divided by the number of ribbons and in that way reduce the effectiveness of ephaptic feedback. The essential point in this discussion is whether the potential distribution within the extracellular space of the cone pedicle will affect the feedback responses. Dmitriev and Mangel [3] argued that each synaptic ribbon should be treated as isolated from each other and that therefore the total potential change induced by ephaptic feedback should be divided by the number of ribbons. In other words they argued that the ephaptic mechanism could only generate large enough responses if the synaptic terminal is iso-potential. Below it will be shown that iso-potentiality is not a critical factor in the model.

When the retina is illuminated evenly, every horizontal cell has $k$identical dendrites that sense the same $[Glu]$. In this case within a cone terminal there will be no current flow from one dendrite to another. Therefore each dendrite can be considered as a single unit each with a conductance of $\frac{1}{k}g_{K}$. For full-field illumination, the difference between dendrites in a particular cone synaptic terminal originating from one horizontal cell or from different horizontal cells can be ignored. Next we considered whether the model will behave differently when the tips of the dendrites would be isolated from each other or when the whole extracellular space in the cone synapse would be isopotential. First the following resistances will be defined:

(3)

For the dendrite that results in the following relations:

$\begin{matrix} R_{dend,K}=R_{HC,K}\cdot k \\ R_{dend,hemi}=R_{HC,hemi}\cdot k \\ R_{dend,Glu}=R_{HC,Glu}\cdot k \end{matrix}$ (4)

and

(5)

Two extreme cases will be analyzed: One in which the tips are fully isolated from each other: the isolated case and one where the dendritic tips are all ending in an isopotential space: the isopotential case. In the isopotential case all dendrites can be considered as one big dendrite. In this condition the following relations hold.

$\begin{matrix} R_{unit,K}=\frac{R_{dend,K}}{l}=\frac{k\cdot R_{HC,K}}{l} \\ R_{unit,hemi}=\frac{R_{dend,hemi}}{l}=\frac{k\cdot R_{HC,hemi}}{l} \\ R_{unit,Glu}=\frac{R_{dend,Glu}}{l}=\frac{k\cdot R_{HC,Glu}}{l} \end{matrix}$ (6)

and

$\begin{matrix} g_{unit,K}=g_{dend,K}\cdot l=\frac{g_{HC,K}\cdot l}{k} \\ g_{unit,hemi}=g_{dend,hemi}\cdot l=\frac{g_{HC,hemi}\cdot l}{k} \\ g_{unit,Glu}=g_{dend,Glu}\cdot l=\frac{g_{HC,Glu}\cdot l}{k} \end{matrix}$ (7)

With , $l$ is the number of dendritic processes invaginating a cone pedicle and the estimated value of $R_{unit,ext}$= 60 MΩ [2] this give for the extracellular resistance related to each dendrite:

$\begin{matrix} R_{unit,hemi}=r\cdot60\text{ M}\Omega\\ R_{dend,hemi}=R_{unit,hemi}\cdot l=r\cdot l\cdot60\text{ M}\Omega\end{matrix}$ (8)

Since, in the isolated case each model horizontal cell unit represents one dendrite, the following relations hold:

$\begin{matrix} R_{unit,K}=R_{dend,K}=R_{HC,K}\cdot k \\ R_{unit,hemi}=R_{dend,hemi}=R_{HC,hemi}\cdot k \\ R_{unit,Glu}=R_{dend,Glu}=R_{HC,Glu}\cdot k \end{matrix}$ (9)

and

$\begin{matrix} g_{unit,K}=\frac{g_{HC,K}}{k} \\ g_{unit,hemi}=\frac{g_{HC,hemi}}{k} \\ g_{unit,Glu}=\frac{g_{HC,Glu}}{k} \end{matrix}$ (10)

In this case one needs to assume that $R_{unit,ext}=l\cdot\text{60 M}\Omega$, since the estimated resistance needs to be distributed over all the dendrites entering one cone. Substitutingin equations (5) and (6) leads to:

$R_{unit,hemi}=r\cdot R_{unit,ext}=r\cdot l\cdot\text{60 M}\Omega$ (11)

Since in this case $R_{unit,hemi}=R_{dend,hemi}$we obtain a similar relation as Equ. (4) showing that the isolated case and the iso-potential case do not differ.

### Scaling

So far we have defined the model in relative values. The only absolute value is $g_{K}$. The next step is to scale all the other variables in the model relative to $g_{K}$such that the model will generate quantitative data. To account for the morphology we need to incorporate one additional scaling factor: a factor to accommodate the connectivity between cones and horizontal cells. There is evidence that, on average, a single horizontal cell contacts 20 cones [12]. However, based on the hexagonal organization of the horizontal cell network, and the presence of three layers of horizontal cells, we estimate that L- and M-cones make contact with about 9 horizontal cells. Hence, to describe the interaction with one cone, the values for conductances and currents in the horizontal cell model (Fig 21A) should be scaled by a factor of about ½ (horizontal cell contacts per cone/cones per horizontal cells). This factor appears as a scaling factor in equation 6). Note that this scaling does not apply to $g_{ext}$as the estimated values for the intersynaptic conductance already corresponds to one cone.

The number of synaptic ribbons per cone pedicle is not included in the model. Dmitriev and Mangel [3] argued that the synaptic ribbons within the cone pedicle are relatively isolated from each other, and therefore the effect of ephaptic feedback should be divided by the number of synaptic ribbons. If one does so, the intersynaptic conductance$g_{ext}$ should also be divided by the number of synaptic ribbons, thus resulting in an identical scaling of all conductances and currents in the model. This would not lead to changes in $V_{HC}$and $V_{ext}$.

### Target values for potentials

$V_{HC}$is determined by the parameters $g_{hemi}$,$g_{Glu}$,$g_{K}$,$g_{ext}$and$E_{K}$. The values of $g_{hemi}$,$g_{Glu}$and$E_{K}$ are unknown, whereas estimates of $g_{K}$ and $g_{ext}$ are available [1,11]. Three experimental observations allow us to estimate the three unknown parameters. (i) In the dark, with both $g_{hemi}$and$g_{Glu}$conducting, horizontal cells rest at -34.7 mV. (ii) When the glutamate gated channels are blocked, the cell hyperpolarizes to -71.4 mV, and (iii) if, in addition, the hemichannels are blocked, the horizontal cell hyperpolarizes to -82.7 mV [2]. Calculating the associated conductances yields the values presented in Table S2

What is the physiological value for the shift of the Ca^2+^-current? In the present study it was shown that -52.7 mV polarization of horizontal cells leads to a shift of the Ca^2+^-current of -9.3 mV. Since the shift depends linearly on the membrane potential [14], one expects that polarizing a horizontal cell from its dark resting membrane potential of -34.1 mV to the maximal light induced potential of -71.4 mV yields a shift of the Ca^2+^-current of -6.6 mV. As we will show, a shift of this magnitude can be obtained by substituting reasonable values for the two free parameters, $p$and $T$.

Two free parameters, for which no precise values are currently available, are still present in the model: $p$, the fraction of glutamate gated channels on the tip of the horizontal cell dendrites, and $T$, the tortuosity factor which describes the increase in diffusion constant due to the properties of the extracellular space. Such factor is around 1.5 [4,5]. The influence of $p$and $T$ on the behavior of the model is evaluated next.

With $p$= 0.75 (75% of the glutamate receptors in the synaptic terminal) and $T$= 1 (no tortuosity), the hyperpolarization of the horizontal cell leads to $\Delta V_{ext}$= -5.12 mV (Table S2), which closely approximates the physiological value. Increasing $T$to 1.5, a value often used in the literature [4,5], resulted in $\Delta V_{ext}$= -8.35 mV (Table S2), a value considerably greater than the change in $V_{ext}$found experimentally. To obtain the physiological value of –6.6 mV required that $T$ be adjusted to 1.24. Fig 12B shows the effect of changing $p$from 0 to 1 on $\Delta V_{ext}$. With higher values of $p$(more receptors in the synaptic terminal), $\Delta V_{ext}$becomes smaller and with lower values of $p$(more receptors in the neuropil), $\Delta V_{ext}$becomes larger. However, it is important to recognize that $\Delta V_{ext}$remains negative even when all glutamate receptors are located diffusely in the synaptic terminal ($p$=1). Although no unique solution exists for $p$and $T$, we have chosen to use $p$= 0.75 and $T$= 1.24 for the simulations presented in this appendix. These are by no means extreme values.

## REFERENCES

1. Vandenbranden CA, Verweij J, Kamermans M, Muller LJ, Ruijter JM, Vrensen GF, Spekreijse H (1996) Clearance of neurotransmitter from the cone synaptic cleft in goldfish retina. Vision Res 36: 3859-3874.

2. Kamermans M, Fahrenfort I, Schultz K, Janssen-Bienhold U, Sjoerdsma T, Weiler R (2001) Hemichannel-mediated inhibition in the outer retina. Science 292: 1178-1180.

3. Dmitriev AV, Mangel SC (2005) Electrical feedback in the cone pedicle: a computational analysis. J Neurophysiol 95: 1419-1427.

4. Rusakov DA, Kullmann DM (1998) Geometric and viscous components of the tortuosity of the extracellular space in the brain. Proc Natl Acad Sci U S A 95: 8975-8980.

5. Nicholson C, Sykova E (1998) Extracellular space structure revealed by diffusion analysis. Trends Neurosci 21: 207-215.

6. Nicholson C, Phillips JM (1981) Ion diffusion modified by tortuosity and volume fraction in the extracellular microenviroment of the rat cerebellum. J Physiol (Lond ) 321: 225-257.

7. Janssen-Bienhold U, Schultz K, Gellhaus A, Schmidt P, Ammermuller J, Weiler R (2001) Identification and localization of connexin26 within the photoreceptor-horizontal cell synaptic complex. Vis Neurosci 18: 169-178.

8. Shields CR, Klooster J, Claassen Y, Mahboob Ul-Hussain, Zoidl G, Dermietzel R, Kamermans M (2007) Retinal Horizontal Cell-Specific Promotor Activity and Protein Expression of Zebrafish Connexin 52.6 & connexin 55.5. J Comp Neurol 501: 765-779.

9. Fahrenfort I, Klooster J, Sjoerdsma T, Kamermans M (2004) The involvement of glutamate-gated channels in negative feedback from horizontal cells to cones. Prog Brain Res 147.

10. Tian M, Chen L, Xie JX, Yang XL, Zhao JW (2003) Expression patterns of inwardly rectifying potassium channel subunits in rat retina. Neurosci Lett 345: 9-12.

11. Dong CJ, Werblin FS (1995) Inward rectifying potassium conductance can accelerate the hyperpolarizing response in retinal horizontal cells. J Neurophysiol 74: 2258-2265.

12. Stell WK, Lightfoot DO (1975) Color-specific interconnections of cones and horizontal cells in the retina of the goldfish. J Comp Neurol 159: 473-502.

13. Yagi T, Kaneko A (1988) The axon terminal of goldfish retinal horizontal cells: A low membrane conductance measured in solitary preparations and its implication to the signal conduction from the soma. J Neurophysiol 59: 482-494.

14. Kraaij DA, Spekreijse H, Kamermans M (2000) The open- and closed-loop gain-characteristics of the cone/horizontal cell synapse in goldfish retina. J Neurophysiol 84: 1256-1265.
