# Supplementary material for: Hemichannel-Mediated and pH-Based Feedback from Horizontal Cells to Cones in the Vertebrate Retina
Source: PLoS One. 2009 Jun 30;4(6):e6090. doi: 10.1371/journal.pone.0006090 (PMC2699542; doi:10.1371/journal.pone.0006090)
Supplement: Table S1 — (0.05 MB DOC) [file pone.0006090.s002.doc]

Supplementary Table 1. Values used to calculate the effective surface area of horizontal cells *in situ*.

|  | **H1** | **H2** | **H3** |
| --- | --- | --- | --- |
| **Soma** |  |  |  |
| Surface (μm^2^) | 618 | 424 | 373 |
| **Dendrites** |  |  |  |
| Number of contacted cones | 23 | 17 | 16 |
| Cones per dendrite | 3 | 3 | 3 |
| Number of dendrites | 8 | 6 | 5 |
| Length (μm) | 23 | 28 | 49 |
| Diameter (μm) | 0.5 | 0.5 | 0.5 |
| Surface (μm^2^) | 283 | 264 | 387 |
| **Axon** |  |  |  |
| Length (μm) | 500 | 500 | 500 |
| Diameter (μm) | 1 | 1 | 1 |
| Surface (μm^2^) | 1571 | 1571 | 1571 |
| **Axon terminal** |  |  |  |
| Length (μm) | 200 | 200 | 200 |
| Diameter (μm) | 6 | 6 | 6 |
| Surface (μm^2^) | 3770 | 3770 | 3770 |
| **Surface** |  |  |  |
| Total surface in situ (μm^2^) | 6242 | 6029 | 6100 |
| Effective surface in situ (μm^2^) | 2660 | 2448 | 2519 |
| Surface of dissociated cells (μm^2^) | 845 | 636 | 682 |
| Surface ratio | 3.1 | 3.9 | 3.7 |
