# Supplementary material for: Hemichannel-Mediated and pH-Based Feedback from Horizontal Cells to Cones in the Vertebrate Retina
Source: PLoS One. 2009 Jun 30;4(6):e6090. doi: 10.1371/journal.pone.0006090 (PMC2699542; doi:10.1371/journal.pone.0006090)
Supplement: Table S2 — (0.04 MB DOC) [file pone.0006090.s003.doc]

Supplementary Table 2 Parametric values used for the simulations

|  | $T$ | $p$ | $E_{K}$ | $g_{K}\text{ at -34.7 mV}$ | $g_{K}\text{ at -71.4 mV}$ | $g_{hemi}$ | $g_{Glu,dark}\text{ at -34.7 mV}$ | $\Delta V_{ext}$ |
| --- | --- | --- | --- | --- | --- | --- | --- | --- |
| 60 MΩ | 1 | 0.75 | -82.7 mV | 6.28 nS | 23.28 nS | 4.73 nS | 6.46 nS | -5.01 mV |
| 74.4 MΩ | 1.24 | 0.75 | -82.7 mV | 6.28 nS | 23.28 nS | 5.07 nS | 6.87 nS | -6.67 mV |
| 90 MΩ | 1.5 | 0.75 | -82.7 mV | 6.28 nS | 23.28 nS | 5.51 nS | 7.33 nS | -8.60 mV |
